# Per- and Polyfluoroalkyl Substances and Papillary Thyroid Carcinoma: An Integrative Study of Bioinformatics, Epidemiological Associations, and In Vitro Responses

**DOI:** 10.3390/ijms27188296

**Published:** 2026-09-17

**Authors:** Yuxin Yan, Hongguang Sun, Meili Cong, Xinxin Qi, Shilei Zhang, Tao Liu

**Affiliations:** School of Public Health, Xinjiang Medical University, Urumqi 830054, China

**Keywords:** per- and polyfluoroalkyl substances, papillary thyroid carcinoma, m7G-associated RNA regulation, PFAS mixtures, EIF4E, NCBP1

## Abstract

Per- and polyfluoroalkyl substances (PFAS) have been associated with thyroid dysfunction, but their relationship with papillary thyroid carcinoma (PTC) and relevant molecular processes remains unclear. We integrated bioinformatics analyses, a hospital-based case–control study, and in vitro experiments to investigate associations between PFAS exposure and PTC and to explore potentially relevant post-transcriptional RNA regulatory processes. Transcriptomic data, PFAS-associated genes, and m^7^G-associated genes were integrated to identify candidate genes. Among 24 overlapping candidates, 21 remained differentially expressed between PTC and normal thyroid tissues after false discovery rate correction, and EIF4E, NCBP1, and AGO2 were identified as candidate hub genes. The case–control study included 60 patients with newly diagnosed PTC and 60 controls. Serum concentrations of 17 PFAS were measured, and individual and mixture associations with PTC status were evaluated using logistic regression, weighted quantile sum regression, quantile g-computation, and Bayesian kernel machine regression. Higher concentrations of several PFAS were associated with lower odds of PTC, although the findings were not fully consistent across analytical models. In vitro, high concentrations of PFOA and PFOS reduced relative thyroid-cell viability, whereas wound-healing responses were variable and did not show a consistent enhancement. PFOA and PFOS exposure was also accompanied by altered EIF4E and NCBP1 mRNA expression, respectively. Overall, these findings support an association between PFAS exposure and PTC and identify m^7^G-associated post-transcriptional regulatory candidates, particularly EIF4E and NCBP1, that may contribute to PFAS-related thyroid cellular responses and warrant further mechanistic investigation.

## 1. Introduction

Papillary thyroid carcinoma (PTC), accounting for approximately 80–90% of all thyroid cancers, is the most common endocrine malignancy, and its incidence has increased steadily worldwide over recent decades [1]. Although most patients with PTC have favorable clinical outcomes, the increasing disease burden, together with recurrence, metastasis, and treatment resistance in a subset of patients, underscores the need to identify additional factors involved in thyroid carcinogenesis. In addition to established risk factors, including ionizing radiation [2], genetic susceptibility [3], and iodine nutritional status [4], growing evidence suggests that environmental pollutants may contribute to the development of PTC. Characterizing molecular responses to environmental exposures may therefore improve our understanding of the environmental etiology and molecular mechanisms of PTC.

Per- and polyfluoroalkyl substances (PFAS) are a large class of persistent synthetic fluorinated chemicals widely used in food packaging, textiles, water-resistant materials, firefighting foams, and numerous industrial and consumer products. Owing to the exceptional stability of their carbon–fluorine bonds, PFAS are highly resistant to environmental degradation and have become ubiquitous environmental contaminants [5,6]. Humans are chronically exposed to PFAS through multiple pathways, including drinking water, diet, air, and consumer products, resulting in their persistence in blood and tissues. Several PFAS have biological half-lives of several years in humans, raising concerns regarding their bioaccumulation and potential adverse health effects [7].

The thyroid gland is an important endocrine target of PFAS exposure. Previous studies suggest that PFAS can bind to serum proteins [8] and thyroid hormone transport proteins [9,10] and may interfere with iodine uptake, thyroid hormone synthesis [11], transport, and metabolism, potentially disrupting thyroid hormone homeostasis. PFAS exposure may also affect thyroid-cell function through oxidative stress, inflammatory responses, nuclear receptor signaling, mitochondrial dysfunction, and metabolic dysregulation [12,13,14]. Epidemiological studies have reported associations between PFAS exposure and altered thyroid hormone concentrations or thyroid diseases; however, evidence regarding thyroid cancer, particularly PTC, remains limited and inconsistent [15,16,17,18,19]. Moreover, the molecular pathways potentially linking PFAS exposure to PTC remain poorly characterized. Toxicogenomic databases and computational target-prediction approaches provide complementary information for characterizing molecular targets potentially associated with environmental chemicals. The Comparative Toxicogenomics Database (CTD) integrates reported chemical–gene and chemical–protein relationships derived from experimental and literature evidence, whereas SwissTargetPrediction predicts potential molecular targets based on chemical structural similarity and ligand–target information. Integrating these two sources can broaden the molecular coverage of PFAS-associated genes and facilitate the identification of candidate regulatory molecules potentially relevant to thyroid carcinogenesis.

Because humans are simultaneously exposed to multiple PFAS, mixture assessment is important for understanding their potential health implications. Individual PFAS are often strongly correlated and may exhibit additive, synergistic, or antagonistic effects, as well as nonlinear exposure–response relationships [20,21]. Consequently, conventional single-pollutant models may not fully capture real-world exposure patterns. Combining conventional regression with mixture-modeling approaches, including weighted quantile sum (WQS) regression, quantile g-computation (qgcomp), and Bayesian kernel machine regression (BKMR), enables the evaluation of overall mixture associations, the relative contributions of individual compounds, and potential nonlinear or interactive relationships with PTC [22,23,24].

Post-transcriptional RNA regulation is an important biological layer through which environmental exposures can reshape cellular adaptation and tumor-related phenotypes [25,26]. PFAS exposure has been associated with oxidative stress, inflammatory signaling, mitochondrial dysfunction, and metabolic disturbance, all of which can influence RNA processing, stability, translation, and cellular stress responses. These effects provide a biological basis for investigating whether PFAS-responsive molecular networks may converge on post-transcriptional RNA regulatory processes. N^7^-methylguanosine (m^7^G) is a defining component of the 5′ cap of eukaryotic messenger RNA (mRNA) and is also present in transfer RNA (tRNA), ribosomal RNA (rRNA), and other RNA species [27]. m^7^G-associated regulatory processes participate in RNA stability, processing, nuclear export, and translation [28,29]. Dysregulation of m^7^G-associated pathways has been implicated in tumor-cell growth, stress adaptation, migration, and remodeling of the tumor immune microenvironment in several malignancies [30,31,32]. Therefore, exploring the intersection between PFAS-associated molecular targets and m^7^G-related regulatory genes may provide insight into post-transcriptional mechanisms involved in PTC.

Among proteins involved in mRNA cap recognition or broader post-transcriptional regulation, eukaryotic translation initiation factor 4E (EIF4E), nuclear cap-binding protein subunit 1 (NCBP1), and Argonaute 2 (AGO2) have distinct but related roles. EIF4E recognizes the mRNA 5′ cap and facilitates cap-dependent translation, including the translation of transcripts associated with cell survival and tumor progression [33]. NCBP1 is a core component of the nuclear cap-binding complex and participates in nascent RNA processing, RNA quality control, transcript stability, and nuclear export [34,35]. By contrast, AGO2 is not a direct mRNA cap-binding protein but functions as the catalytic component of the RNA-induced silencing complex, mediating microRNA-dependent post-transcriptional gene silencing [36]. Together, these functions place EIF4E, NCBP1, and AGO2 at key nodes of RNA cap recognition, RNA processing, translational control, and post-transcriptional gene regulation, supporting their relevance as candidate molecules for investigating PFAS-associated molecular responses in PTC. Altered expression of these genes, however, does not directly indicate changes in intracellular m^7^G modification levels.

To address these knowledge gaps, we integrated bioinformatics analyses, a hospital-based case–control study, and in vitro experiments using a sequential framework comprising candidate-gene screening, epidemiological association analysis, and targeted experimental evaluation. Candidate genes potentially linking PFAS exposure to m^7^G-associated post-transcriptional regulation were identified by integrating thyroid cancer transcriptomic data, PFAS-associated genes, and m^7^G-associated gene sets. Associations of individual PFAS and PFAS mixtures with PTC case status were then evaluated using conventional regression and multiple mixture-modeling approaches. Finally, representative PFAS and candidate genes were selected for in vitro evaluation of thyroid-cell viability, wound closure, and gene-expression responses. Through this integrated framework, the study aimed to characterize PFAS–PTC associations and identify molecular candidates that may participate in PFAS-responsive post-transcriptional regulation in thyroid cells.

## 2. Results

### 2.1. Identification of PFAS-Associated m^7^G Candidate Genes

**Figure 1:**
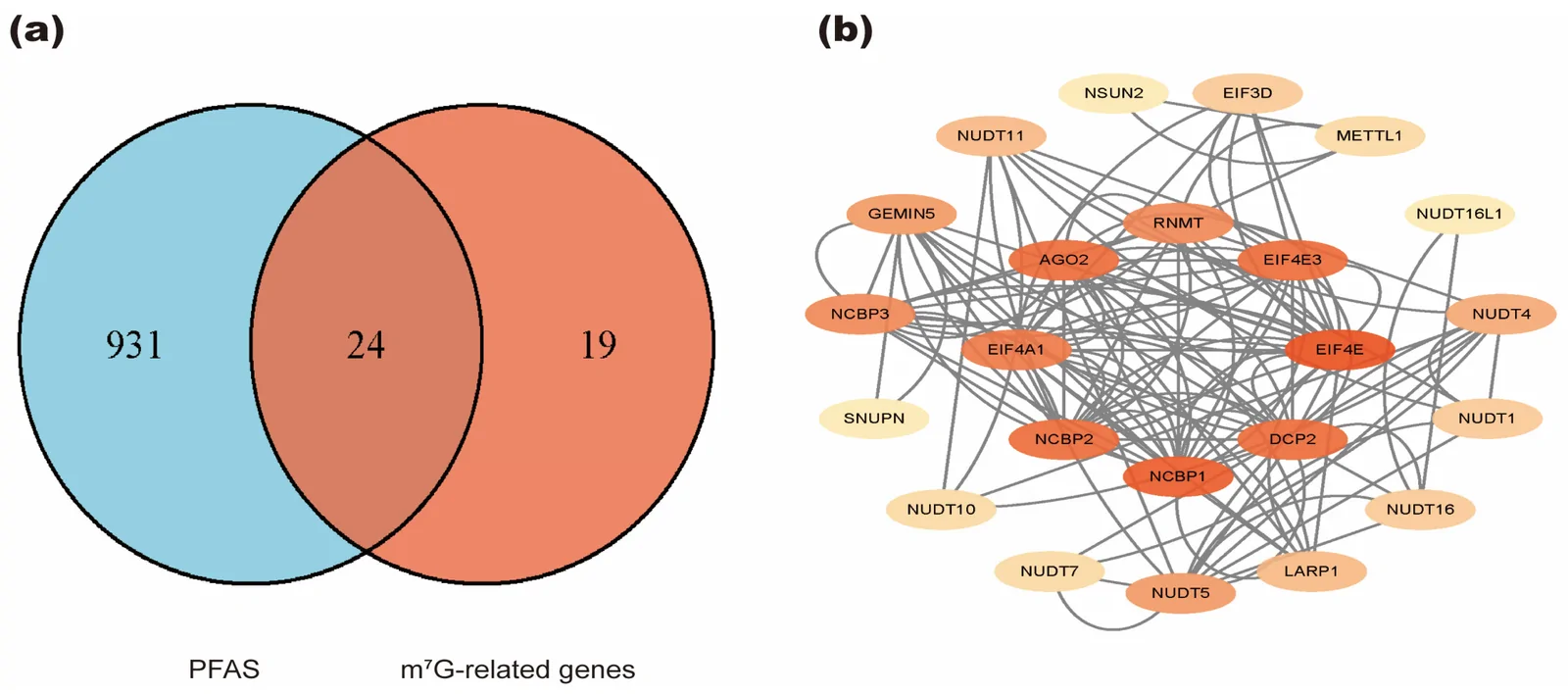
Identification of PFAS-associated m^7^G candidate genes and construction of the protein–protein interaction network. (**a**) Venn diagram showing the intersection of the PFAS-associated and m^7^G-associated gene sets, yielding 24 overlapping candidate genes; (**b**) protein–protein interaction network constructed from the 24 overlapping candidate genes.

To identify genes shared between the PFAS-associated and m^7^G-associated gene sets, the two gene sets were intersected. After gene-symbol standardization and duplicate removal, 955 unique PFAS-associated genes and 43 unique m^7^G-associated genes were retained, yielding 24 overlapping candidate genes (Figure 1a). A protein–protein interaction (PPI) network constructed from these candidates showed multiple reported or predicted protein associations (Figure 1b).

**Figure 2:**
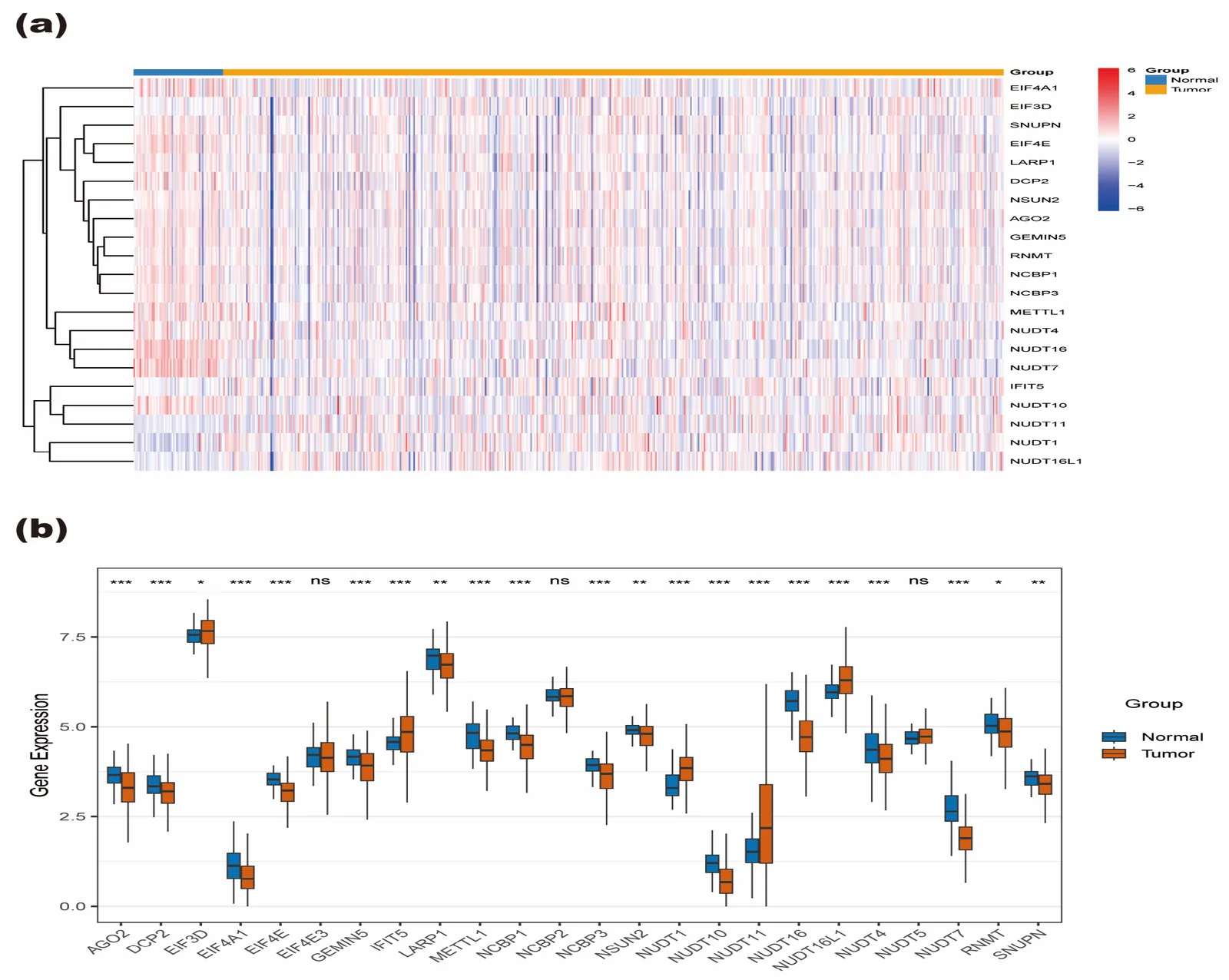
Expression profiles of PFAS-associated m^7^G candidate genes in papillary thyroid carcinoma and normal thyroid tissues. (**a**) Heatmap showing the expression profiles of the 24 overlapping candidate genes in papillary thyroid carcinoma and normal thyroid tissues. (**b**) Expression distributions and between-group comparisons of the candidate genes. * *p* < 0.05, ** *p* < 0.01, *** *p* < 0.001; ns, not significant.

Differential expression of the 24 overlapping candidate genes was subsequently evaluated between PTC and normal thyroid tissues using the TCGA-THCA dataset. The hierarchical clustering heatmap illustrated differences in candidate-gene expression patterns between the two tissue groups. After Benjamini–Hochberg false discovery rate correction across the 24 candidate genes, 21 remained significantly differentially expressed (FDR < 0.05; Figure 2a,b and Table S14). Chromosomal localization analysis showed that these 21 candidate genes were distributed across multiple autosomes and the X chromosome, with no candidate genes located on the Y chromosome (Figure S1).

#### 2.1.1. Functional Enrichment of Differentially Expressed Candidate Genes

**Figure 3:**
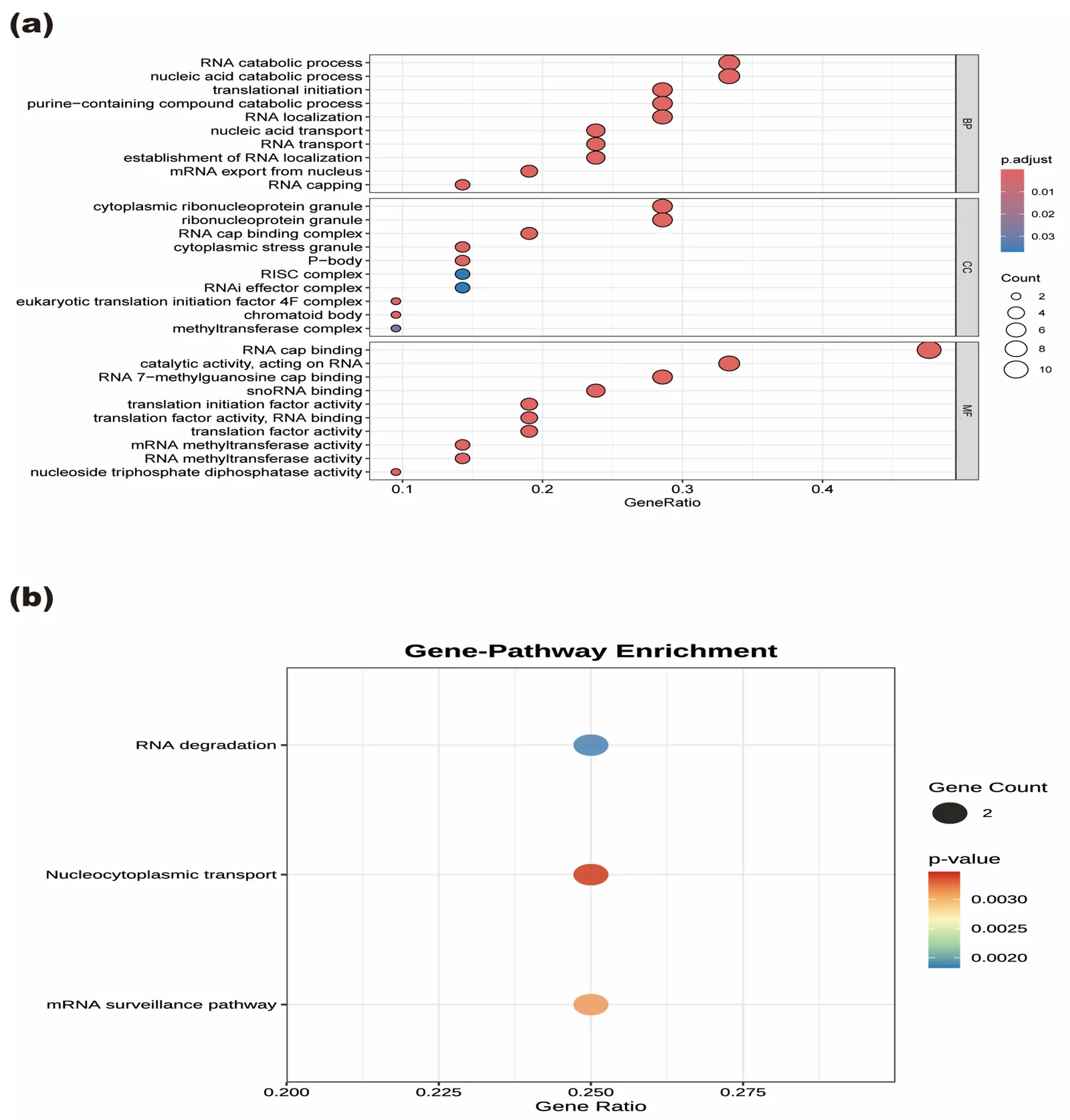
Functional enrichment of differentially expressed PFAS-associated m^7^G candidate genes. (**a**) Gene Ontology enrichment analysis of the 21 differentially expressed candidate genes, including biological process, cellular component, and molecular function categories; (**b**) Kyoto Encyclopedia of Genes and Genomes pathway enrichment analysis.

Gene Ontology (GO) and Kyoto Encyclopedia of Genes and Genomes (KEGG) enrichment analyses were performed for the 21 differentially expressed candidate genes. In the biological process (BP) category, enriched terms included translation initiation, RNA localization, RNA catabolic processes, nucleic acid catabolic processes, and RNA transport. Enriched cellular component (CC) terms included the RNA cap-binding complex, cytoplasmic ribonucleoprotein granules, and ribonucleoprotein granules. In the molecular function (MF) category, enriched terms included RNA cap binding, RNA 7-methylguanosine cap binding, small nucleolar RNA binding, and catalytic activity acting on RNA (Figure 3a).

KEGG pathway analysis identified enrichment in RNA degradation, nucleocytoplasmic transport, and the mRNA surveillance pathway (Figure 3b). These enrichment patterns were consistent with functions related to RNA cap recognition, translation initiation, RNA transport, and RNA metabolism.

#### 2.1.2. PPI Network Topology and Identification of Candidate Hub Genes

**Figure 4:**
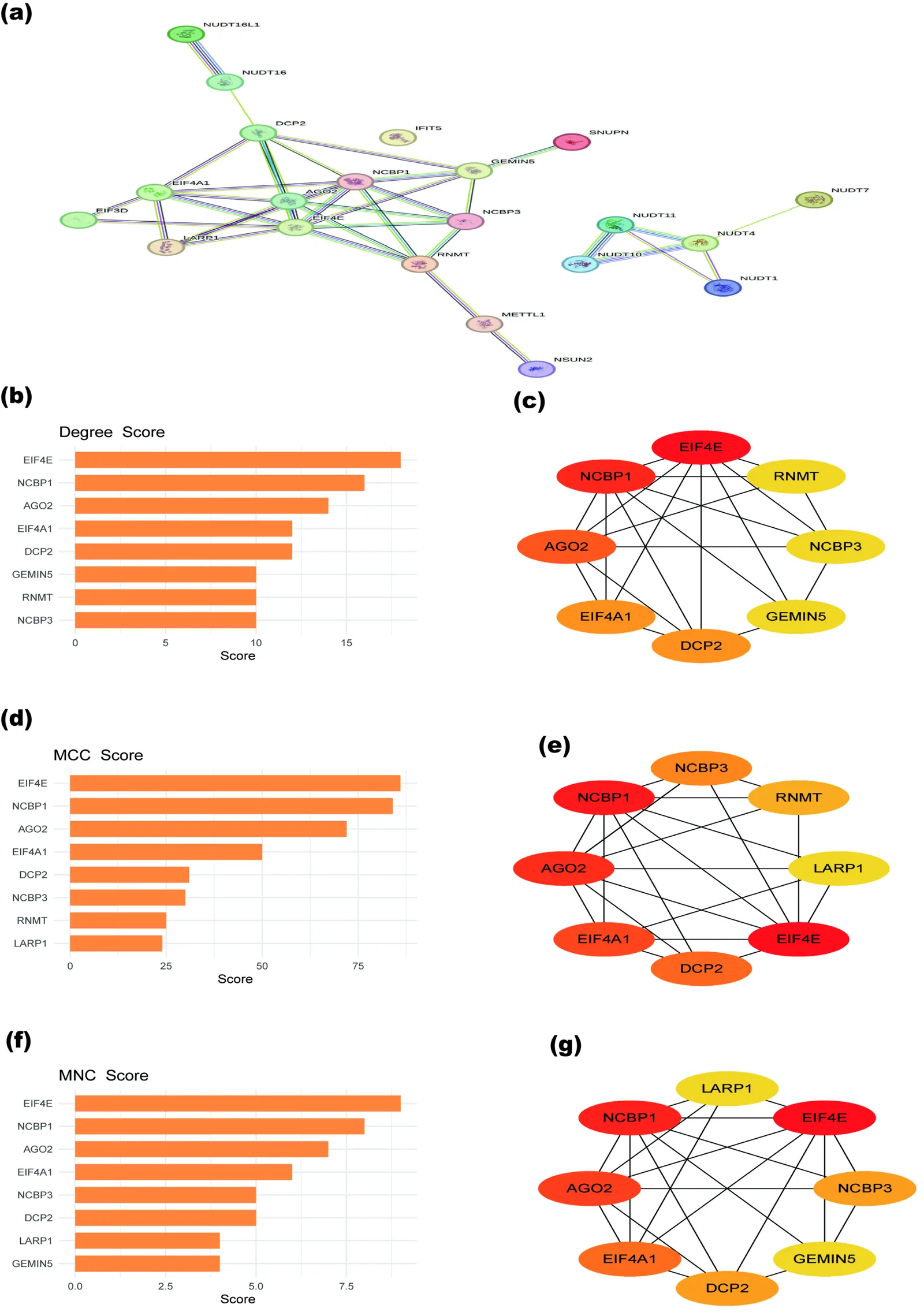
Identification of candidate hub genes based on protein–protein interaction network topology. (**a**) Protein–protein interaction network of the differentially expressed candidate genes. (**b**,**c**) Ranking of candidate genes and the corresponding subnetwork identified using the Degree algorithm. (**d**,**e**) Ranking and corresponding subnetwork identified using the Maximal Clique Centrality algorithm. (**f**,**g**) Ranking and corresponding subnetwork identified using the Maximum Neighborhood Component algorithm.

Three topological algorithms—Degree, Maximal Clique Centrality (MCC), and Maximum Neighborhood Component (MNC)—were applied to rank nodes in the PPI network of the differentially expressed candidate genes. EIF4E, NCBP1, and AGO2 consistently ranked among the leading candidates across all three algorithms and were therefore retained as candidate hub genes based on network topology (Figure 4).

#### 2.1.3. Associations of Candidate Hub-Gene Expression with Estimated Immune-Cell Proportions

**Figure 5:**
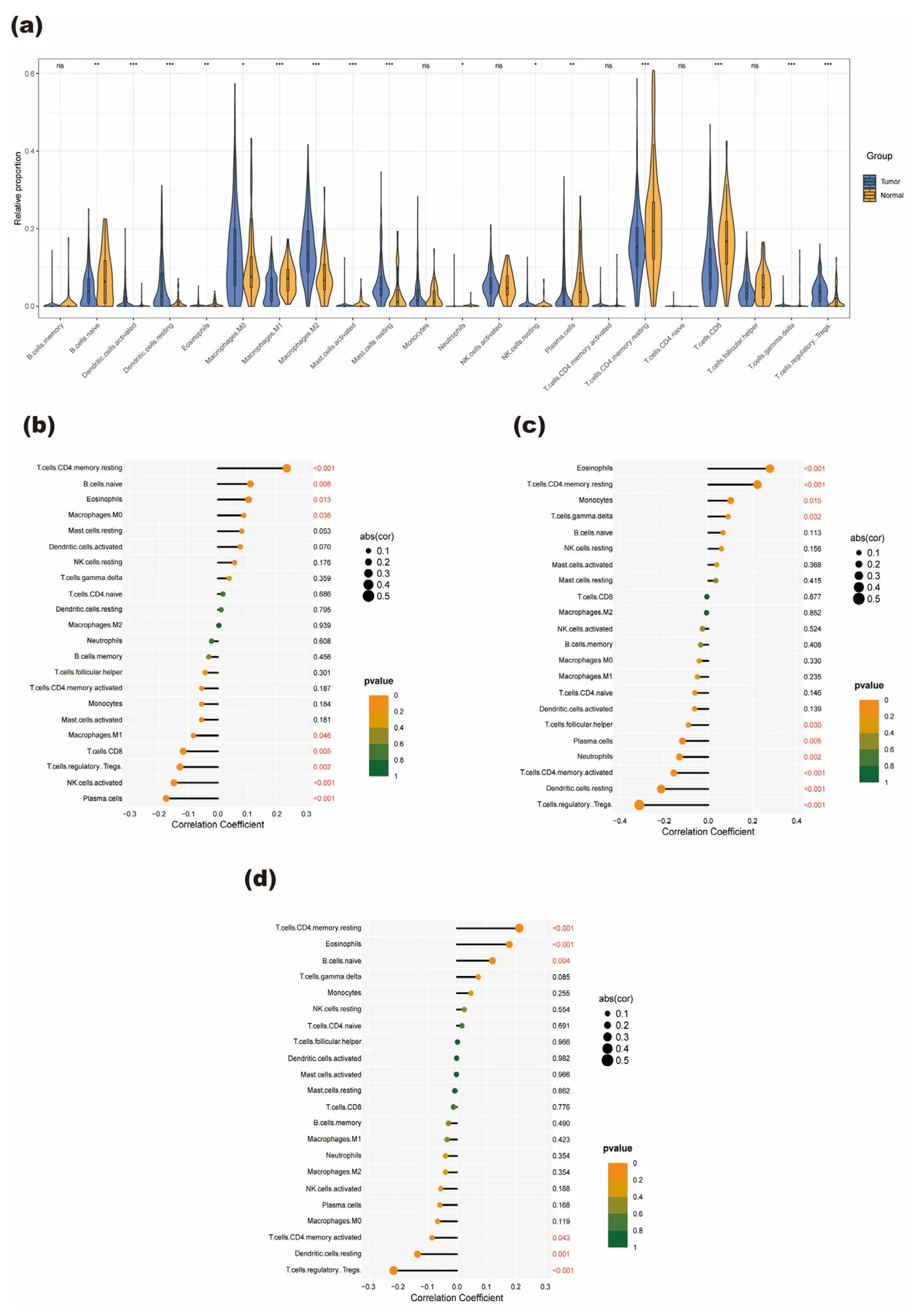
Estimated immune-cell proportions and their correlations with candidate hub-gene expression in papillary thyroid carcinoma. (**a**) Comparison of the estimated relative proportions of 22 immune-cell subsets between papillary thyroid carcinoma and normal thyroid tissues. (**b**) Spearman correlations between AGO2 expression and estimated immune-cell proportions. (**c**) Spearman correlations between EIF4E expression and estimated immune-cell proportions. (**d**) Spearman correlations between NCBP1 expression and estimated immune-cell proportions. * *p* < 0.05, ** *p* < 0.01, *** *p* < 0.001; ns, not significant.

The relative proportions of 22 immune-cell subsets were estimated using the CIBERSORT algorithm. At the nominal significance threshold of *p* < 0.05, the estimated proportions of 16 immune-cell subsets differed between PTC and normal thyroid tissues (Figure 5a).

Spearman correlation analyses showed that AGO2, EIF4E, and NCBP1 expression was associated with the estimated proportions of several immune-cell subsets. EIF4E and NCBP1 expression was positively correlated with the estimated proportions of resting memory CD4^+^ T cells and eosinophils and negatively correlated with those of regulatory T cells and resting dendritic cells. AGO2 expression was correlated with the estimated proportions of resting memory CD4^+^ T cells, plasma cells, natural killer cells, and several other T-cell subsets (Figure 5b–d). These computational associations do not establish direct regulation of immune-cell infiltration by the candidate genes.

Considering the differential expression findings, functional enrichment patterns, PPI-network rankings, and experimental feasibility, EIF4E and NCBP1 were prioritized for targeted in vitro evaluation.

### 2.2. Characteristics of the Study Participants

A total of 120 participants were included in the hospital-based case–control study, comprising 60 PTC cases and 60 controls. Controls were frequency matched to cases by age and sex, and the distributions of these variables did not differ significantly between the two groups. Compared with controls, PTC cases had a higher mean body mass index (25.8 vs. 23.1 kg/m^2^; *p* < 0.001) and were more likely to reside in rural areas (26.7% vs. 8.3%; *p* = 0.008). No significant between-group differences were observed in marital status, smoking status, or alcohol consumption. Participant characteristics are summarized in Table 1.

### 2.3. Serum PFAS Concentrations and Intercorrelations

#### 2.3.1. Detection Frequencies and Concentration Distributions

A total of 17 PFAS were measured in serum samples, of which 14 were detected in at least one participant. PFOS and PFOA were detected in all participants and had the highest median serum concentrations among the measured compounds. In the overall study population, the median PFOS concentration was 1.11 ng/mL (IQR, 0.61–1.76 ng/mL), and the median PFOA concentration was 0.90 ng/mL (IQR, 0.55–1.36 ng/mL). PFUnA and 9Cl-PF3ONS were detected in 98.3% and 91.7% of participants, respectively.

The distributions of serum PFAS concentrations in PTC cases and controls are presented in Table 2. Median concentrations of 9Cl-PF3ONS, PFUnA, PFDA, PFBA, PFTrDA, and PFPeA were lower in PTC cases than in controls (all *p* < 0.05). PFBS concentrations also differed between the two groups; however, PFBS was excluded from subsequent association analyses because of its low detection frequency. No significant between-group differences were observed for PFOS, PFOA, PFHxS, PFDoA, PFHpA, PFNA, or PFTeDA.

#### 2.3.2. Serum PFAS Concentrations Stratified by Sex and BMI

To further characterize serum PFAS concentrations, subgroup analyses were conducted according to sex and BMI. The distributions of serum PFAS concentrations among women, participants with BMI < 24 kg/m^2^, and participants with BMI ≥ 24 kg/m^2^ are presented in Tables S1–S3.

Among women, serum concentrations of 9Cl-PF3ONS, PFBS, PFBA, and PFPeA differed between PTC cases and controls. Among participants with BMI < 24 kg/m^2^, between-group differences were observed for 9Cl-PF3ONS, PFBS, PFOS, and PFBA. Among participants with BMI ≥ 24 kg/m^2^, PFBS, PFUnA, PFBA, PFPeA, and PFTrDA differed between the two groups. Several PFAS showed lower concentrations in PTC cases than in controls within selected subgroups; however, the specific compounds showing statistical significance were not consistent across subgroup analyses. These findings were therefore considered exploratory.

#### 2.3.3. Correlations Among Serum PFAS Components

**Figure 6:**
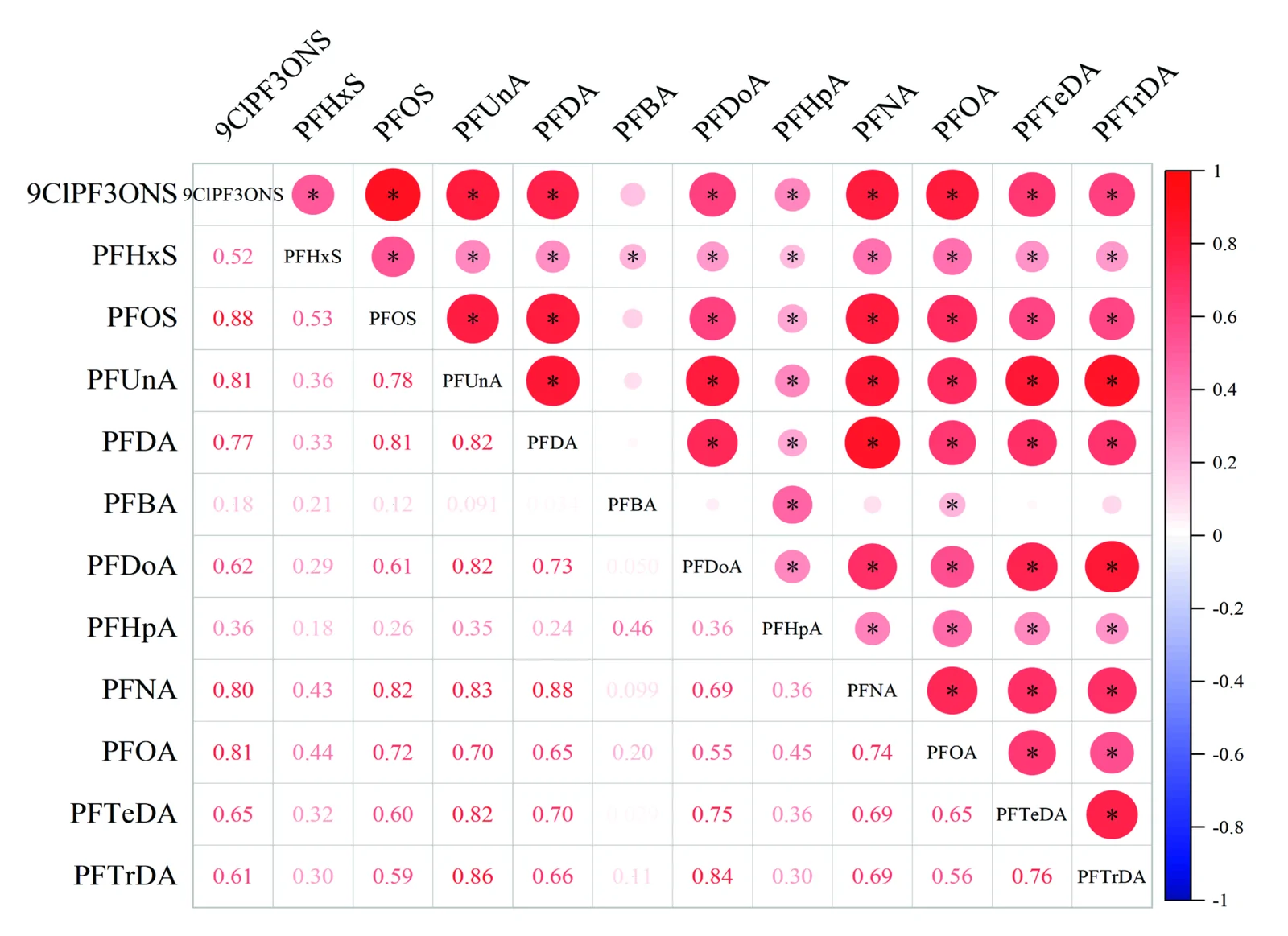
Spearman correlations among serum PFAS concentrations. * *p* < 0.05.

Spearman correlation analysis showed positive correlations of varying magnitude among several serum PFAS compounds, consistent with correlated exposure patterns in the study population (Figure 6). Relatively stronger positive correlations were observed between 9Cl-PF3ONS and PFOS, between PFDA and PFNA, and between PFUnA and PFTrDA. By contrast, PFBA generally showed weaker correlations with the other PFAS compounds.

### 2.4. Associations of Individual PFAS with the Odds of PTC

#### 2.4.1. Logistic Regression Analysis of Continuous PFAS Concentrations

Multivariable logistic regression was used to evaluate associations of individual PFAS with the odds of PTC. Serum PFAS concentrations were natural logarithm-transformed and analyzed without further z-score standardization. Model 1 was unadjusted, whereas Model 2 was adjusted for age, sex, BMI, place of residence, smoking status, and alcohol consumption. Odds ratios represent the change in the odds of PTC associated with a one-unit increase in the ln-transformed serum concentration of each PFAS.

In the adjusted models, 9Cl-PF3ONS, PFOS, PFUnA, PFDA, PFBA, PFNA, and PFTrDA were inversely associated with the odds of PTC (all *p* < 0.05). The adjusted ORs (95% CIs) were 0.48 (0.32–0.74) for 9Cl-PF3ONS, 0.43 (0.23–0.79) for PFOS, 0.42 (0.25–0.69) for PFUnA, 0.53 (0.31–0.90) for PFDA, 0.06 (0.01–0.26) for PFBA, 0.60 (0.37–0.98) for PFNA, and 0.55 (0.34–0.90) for PFTrDA. No statistically significant associations were observed for PFHxS, PFDoA, PFHpA, PFOA, or PFTeDA (Table 3). After Benjamini–Hochberg FDR correction across the 12 PFAS evaluated in the continuous models, the associations for 9Cl-PF3ONS, PFOS, PFUnA, PFDA, PFBA, and PFTrDA remained statistically significant, whereas the association for PFNA did not retain statistical significance after correction.

#### 2.4.2. Logistic Regression Analysis by PFAS Exposure Tertiles

For categorical analyses, the ln-transformed concentration of each PFAS was divided into tertiles using the 33rd and 66th percentiles, corresponding to low-, medium-, and high-exposure groups. The lowest tertile served as the reference category. Results for the overall population and the sex- and BMI-stratified analyses are presented in Tables S4–S7.

In the overall population, participants in the highest tertile of 9Cl-PF3ONS had lower odds of PTC than those in the lowest tertile after covariate adjustment (OR = 0.27, 95% CI: 0.09–0.77). The trend remained statistically significant after false discovery rate correction (pFDR = 0.036). For PFUnA, the adjusted ORs were 0.18 (95% CI: 0.05–0.55) for the middle tertile and 0.22 (95% CI: 0.07–0.70) for the highest tertile, with an FDR-adjusted trend *p* value of 0.025.

For PFBA, the adjusted ORs were 0.22 (95% CI: 0.06–0.72) and 0.10 (95% CI: 0.03–0.32) for the middle and highest tertiles, respectively, and the trend remained significant after FDR correction (pFDR = 0.003). Participants in the highest PFTrDA tertile also had lower odds of PTC than those in the lowest tertile (OR = 0.21, 95% CI: 0.06–0.66), with an FDR-adjusted trend *p* value of 0.036.

The adjusted ORs for the highest tertiles of PFOS and PFDA were 0.31 (95% CI: 0.10–0.92) and 0.32 (95% CI: 0.10–0.95), respectively. However, their trend tests were not statistically significant after FDR correction. For PFTeDA, the adjusted ORs for the middle and highest tertiles were greater than 1, but neither the individual estimates nor the trend test provided clear evidence of an association.

The stratified analyses identified several inverse associations within selected subgroups. However, the PFAS compounds showing statistical significance differed across the sex and BMI strata, and several estimates had wide confidence intervals. The subgroup results were therefore interpreted as exploratory.

#### 2.4.3. Restricted Cubic Spline Analysis

Restricted cubic spline models were used to evaluate potential nonlinear exposure–response associations between ln-transformed serum PFAS concentrations and the odds of PTC. Models were adjusted for age, sex, BMI, place of residence, smoking status, and alcohol consumption.

**Figure 7:**
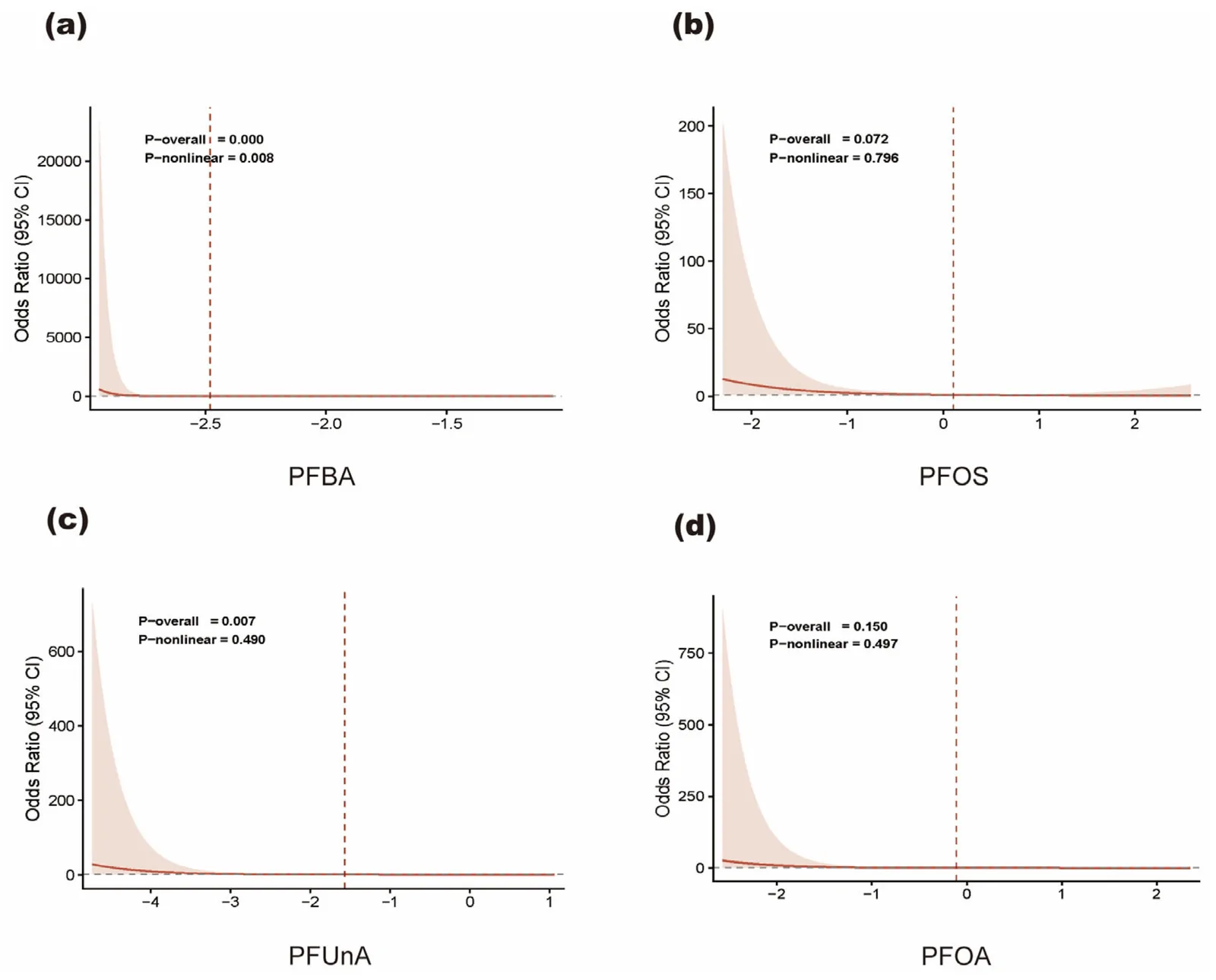
Restricted cubic spline analyses of PFBA, PFOS, PFUnA, and PFOA in relation to the odds of papillary thyroid carcinoma. (**a**) Restricted cubic spline analysis of PFBA in relation to the odds of papillary thyroid carcinoma. (**b**) Restricted cubic spline analysis of PFOS in relation to the odds of papillary thyroid carcinoma. (**c**) Restricted cubic spline analysis of PFUnA in relation to the odds of papillary thyroid carcinoma. (**d**) Restricted cubic spline analysis of PFOA in relation to the odds of papillary thyroid carcinoma.

Evidence of a nonlinear association was observed for PFBA (*p* for nonlinearity = 0.0076). The overall association tests were also statistically significant for 9Cl-PF3ONS, PFUnA, and PFTrDA, but there was no evidence of nonlinearity for these compounds. No statistically significant nonlinear associations were observed for the remaining PFAS. RCS curves for selected PFAS are presented in Figure 7, and results for the other compounds are shown in Figures S2 and S3.

### 2.5. Associations of PFAS Mixtures with the Odds of PTC

Weighted quantile sum (WQS) regression, quantile g-computation (qgcomp), and Bayesian kernel machine regression (BKMR) were used to evaluate associations of PFAS mixtures with the odds of PTC. All models were adjusted for age, sex, BMI, place of residence, smoking status, and alcohol consumption.

**Figure 8:**
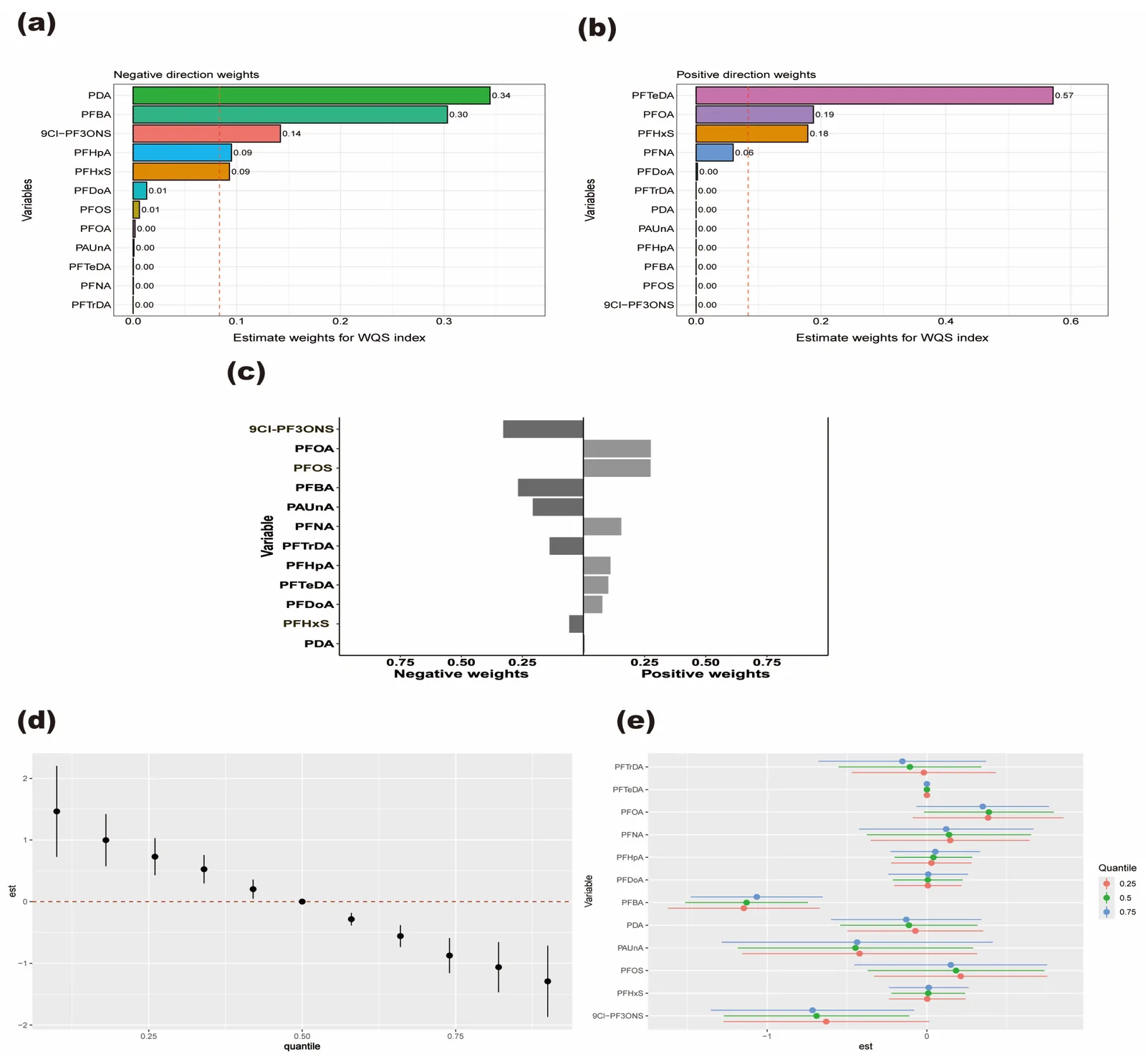
Associations of PFAS mixtures with the odds of papillary thyroid carcinoma estimated using mixture-model approaches. (**a**) Negative-direction component weights estimated using weighted quantile sum regression. (**b**) Positive-direction component weights estimated using weighted quantile sum regression. (**c**) Component weights estimated using quantile g-computation. (**d**) Overall mixture effect estimated using Bayesian kernel machine regression. (**e**) Estimated single-component effects from BKMR with the other PFAS fixed at their 25th, 50th, and 75th percentiles.

WQS regression was performed using separate positive- and negative-direction constraint models. The negative WQS index yielded an OR of 0.43 (95% CI: 0.15–1.22; *p* = 0.114), whereas the positive WQS index yielded an OR of 0.94 (95% CI: 0.39–2.29; *p* = 0.897). Neither association was statistically significant. The component weights are presented in Figure 8a,b, and the subgroup results are shown in Figure S4.

In the qgcomp analysis, a joint one-quartile increase in all PFAS components was associated with lower odds of PTC (OR = 0.16, 95% CI: 0.04–0.61; *p* < 0.05). Positive and negative component weights are presented in Figure 8c, and the subgroup results are shown in Figure S5.

In the BKMR analysis, the condition in which all PFAS components were fixed at their median concentrations was used as the reference. As the joint PFAS exposure percentile increased, the estimated overall mixture association showed a generally inverse pattern. Sensitivity analysis using 50 rather than 10 knots produced a similar overall mixture pattern and broadly comparable PIP rankings. PFBA retained the highest PIP in both specifications, with PIPs of 0.994 and 0.990 in the overall population. The estimated overall mixture association and single-component effects are presented in Figure 8d,e. Subgroup results are shown in Figures S6 and S7, and detailed BKMR effect estimates and posterior inclusion probabilities are presented in Tables S11 and S12.

Overall, WQS regression did not identify a statistically significant mixture association, whereas qgcomp indicated an inverse association between the PFAS mixture and the odds of PTC. BKMR also showed a generally inverse pattern at higher joint exposure percentiles, and the similar results obtained under the 10- and 50-knot specifications supported the stability of the principal BKMR pattern.

### 2.6. Changes in Thyroid-Cell Viability Following PFOA and PFOS Exposure

**Figure 9:**
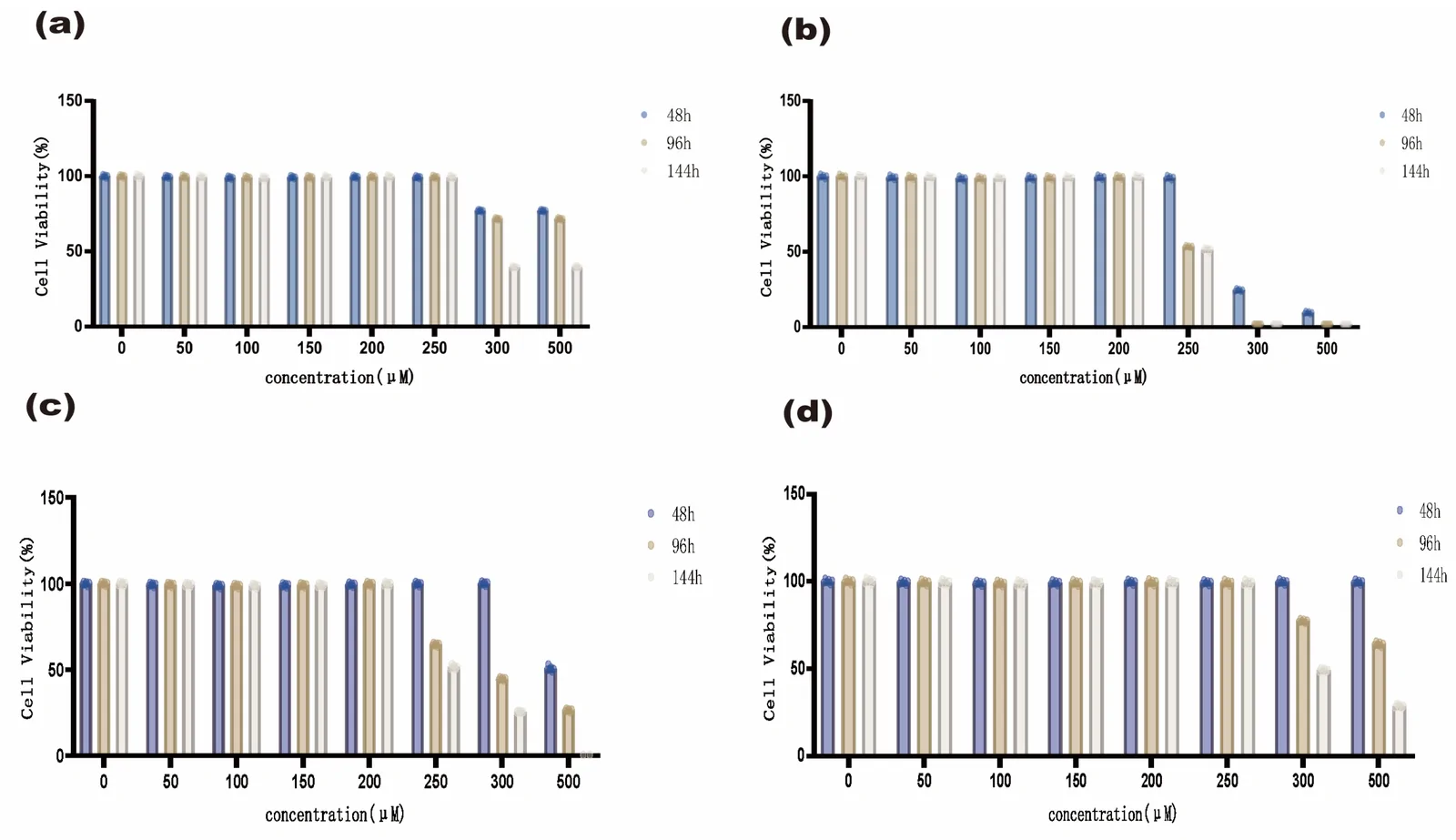
Relative viability of thyroid cells following PFOA and PFOS exposure. (**a**) Relative viability of Nthy-ori 3-1 cells exposed to PFOA. (**b**) Relative viability of Nthy-ori 3-1 cells exposed to PFOS. (**c**) Relative viability of TPC-1 cells exposed to PFOA. (**d**) Relative viability of TPC-1 cells exposed to PFOS.

The viability of human normal thyroid epithelial Nthy-ori 3-1 cells and PTC-derived TPC-1 cells following PFOA or PFOS exposure was evaluated using the CCK-8 assay. Cells were exposed to 0, 50, 100, 150, 200, 250, 300, or 500 μmol/L PFOA or PFOS, and relative cell viability was assessed after 48, 96, and 144 h (Figure 9).

In Nthy-ori 3-1 cells, exposure to 0–200 μmol/L PFOA produced relatively small changes in mean cell viability across the evaluated time points. Lower mean viability was observed at concentrations of 250 μmol/L or higher, with the largest reductions occurring in the 300 and 500 μmol/L groups and after longer exposure durations (Figure 9a). A generally similar pattern was observed following PFOS exposure: mean viability remained relatively stable at 0–200 μmol/L but was lower following exposure to 250–500 μmol/L, particularly at the higher concentrations and longer durations (Figure 9b).

In TPC-1 cells, exposure to 0–200 μmol/L PFOA was associated with relatively small changes in mean cell viability. Lower viability was observed at concentrations of 250 μmol/L or higher, with the largest reductions in the 300 and 500 μmol/L groups after prolonged exposure (Figure 9c). Following PFOS exposure, mean cell viability remained relatively stable at 0–250 μmol/L but was lower in the 300 and 500 μmol/L groups, with more pronounced reductions at longer exposure durations (Figure 9d).

Overall, low-to-moderate concentrations of PFOA and PFOS produced relatively limited changes in the CCK-8 signal, whereas higher concentrations were associated with reduced relative viability in both thyroid cell lines. Concentrations associated with limited loss of cell viability were selected for the subsequent wound-healing and qRT-PCR experiments.

### 2.7. Wound Closure Following PFOA and PFOS Exposure in Thyroid Cells

Wound closure following PFOA or PFOS exposure was evaluated in TPC-1 and Nthy-ori 3-1 cells using a wound-healing assay. Wound areas decreased to varying degrees in all treatment groups between 0 and 48 h. By 48 h, wounds in the control and most exposure groups were nearly completely closed, resulting in relatively small between-group differences. Therefore, the descriptive comparisons below focus primarily on the 24-h wound-closure rates.

#### 2.7.1. Effects of PFOA and PFOS on Wound Closure in TPC-1 Cells

**Figure 10:**
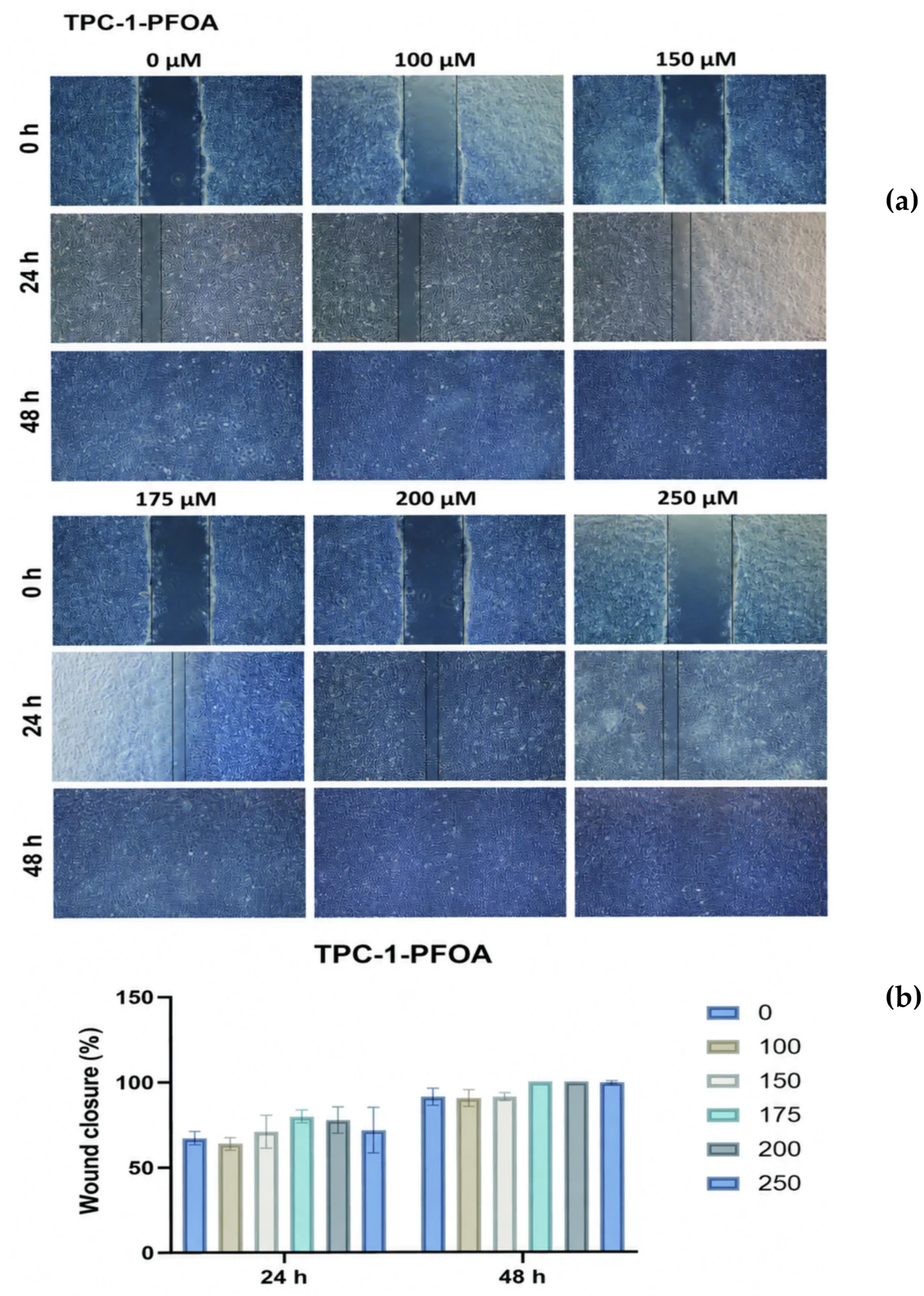
Wound closure in TPC-1 cells following PFOA exposure. (**a**)  Representative wound-healing images of TPC-1 cells exposed to different concentrations of PFOA for 0, 24, and 48 h. (**b**) Quantitative analysis of wound-closure rates in TPC-1 cells treated with different concentrations of PFOA. Quantitative analysis of wound-closure rates following PFOA exposure.

After 24 h of PFOA exposure, the mean wound-closure rates in TPC-1 cells ranged from 63.97% to 79.84%. The mean rate was 67.36% in the untreated control group, 63.97% in the 100 μmol/L group, 71.07–79.84% in the 150–200 μmol/L groups, and 71.81% in the 250 μmol/L group. The highest mean value was observed at 175 μmol/L. However, wound-closure rates fluctuated across the exposure concentrations, and no clear monotonic concentration-related pattern was observed (Figure 10).

**Figure 11:**
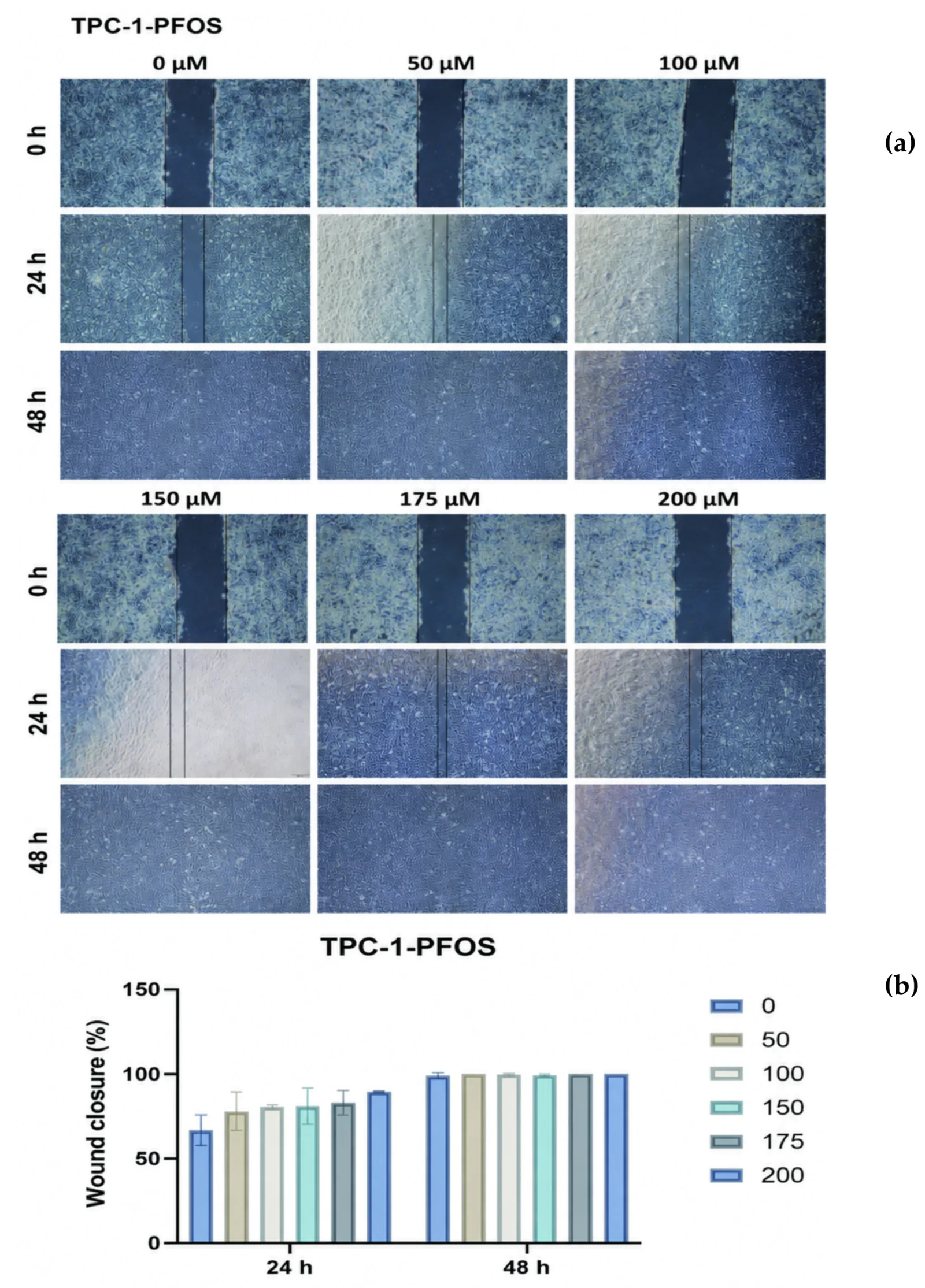
Wound closure in TPC-1 cells following PFOS exposure. (**a**)  Representative wound-healing images of TPC-1 cells exposed to different concentrations of PFOS for 0, 24, and 48 h. (**b**) Quantitative analysis of wound-closure rates following PFOS exposure.

After 24 h of PFOS exposure, the mean wound-closure rate was 66.81% in the untreated control group, ranged from 78.08% to 83.03% in the 50–175 μmol/L groups, and was 89.71% in the 200 μmol/L group. Mean wound-closure rates were numerically higher in the PFOS-treated groups than in the untreated control group, with the highest mean value observed at 200 μmol/L (Figure 11).

#### 2.7.2. Effects of PFOA and PFOS on Wound Closure in Nthy-ori 3-1 Cells

After 24 h of PFOA exposure, the mean wound-closure rates in Nthy-ori 3-1 cells ranged from 52.82% to 85.51%. The mean rate was 52.82% in the untreated control group, ranged from 59.29% to 73.89% in the 100–175 μmol/L groups, was 61.36% in the 200 μmol/L group, and was 85.51% in the 250 μmol/L group. Wound-closure rates varied across concentrations, with no clear monotonic concentration-related pattern (Figure S8).

After 24 h of PFOS exposure, mean wound-closure rates ranged from 77.01% to 90.43%. The mean rate was 85.03% in the untreated control group, 82.00% and 77.01% in the 50 and 100 μmol/L groups, 88.19% and 90.43% in the 150 and 175 μmol/L groups, and 77.50% in the 200 μmol/L group. No consistent concentration-related pattern was observed (Figure S9).

Overall, wound-closure rates varied across PFOA and PFOS concentrations in both cell lines, without a consistent concentration-dependent pattern. Although several PFOS-treated TPC-1 groups had numerically higher mean wound-closure rates at 24 h, this pattern was not consistent across compounds and cell lines.

### 2.8. EIF4E mRNA Expression Following PFOA Exposure and NCBP1 mRNA Expression Following PFOS Exposure

**Figure 12:**
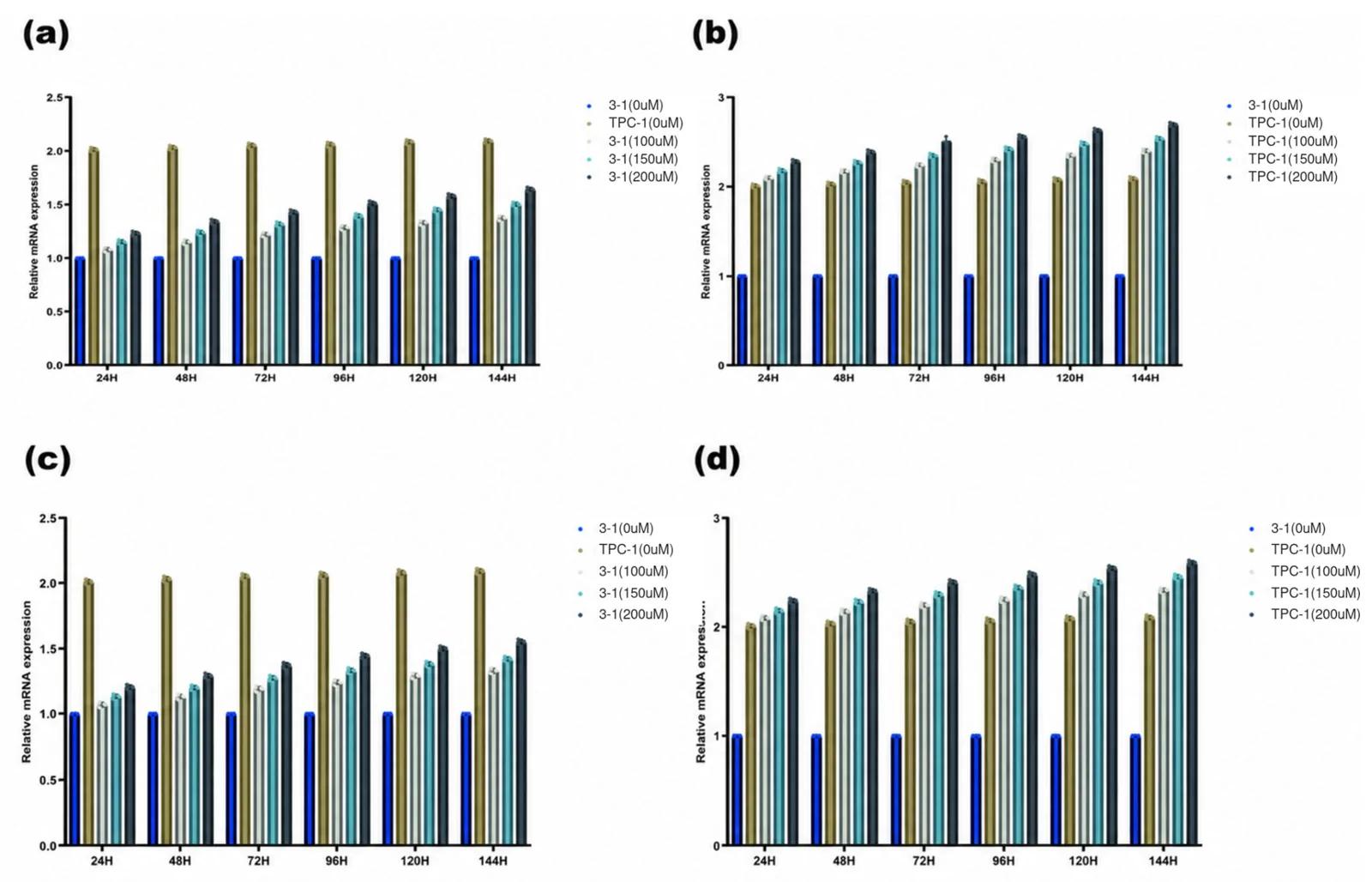
EIF4E and NCBP1 mRNA expression in thyroid cells following PFAS exposure. (**a**) Relative EIF4E mRNA expression in Nthy-ori 3-1 cells following PFOA exposure. (**b**) Relative EIF4E mRNA expression in TPC-1 cells following PFOA exposure. (**c**) Relative NCBP1 mRNA expression in Nthy-ori 3-1 cells following PFOS exposure. (**d**) Relative NCBP1 mRNA expression in TPC-1 cells following PFOS exposure. Expression values are presented relative to untreated Nthy-ori 3-1 cells.

Selected candidate-gene expression responses were evaluated by qRT-PCR in Nthy-ori 3-1 and TPC-1 cells. EIF4E mRNA expression was assessed following PFOA exposure, whereas NCBP1 mRNA expression was assessed following PFOS exposure. Cells were exposed to 0, 100, 150, or 200 μmol/L of the corresponding PFAS for 24, 48, 72, 96, 120, or 144 h (Figure 12).

#### 2.8.1. Changes in EIF4E mRNA Expression Following PFOA Exposure

In Nthy-ori 3-1 cells, mean EIF4E mRNA expression was generally higher in the PFOA-treated groups than in the corresponding untreated controls. Expression tended to be higher after longer exposure durations, and the 200 μmol/L group generally showed the highest mean expression. However, the magnitude of the response varied across individual concentrations and time points (Figure 12a).

In TPC-1 cells, mean EIF4E mRNA expression also generally increased with exposure duration following PFOA treatment. Higher mean expression was often observed at the higher PFOA concentrations, with the 200 μmol/L group generally showing the highest values. Across the evaluated exposure groups, the highest mean expression values were observed at 144 h (Figure 12b).

Overall, PFOA-treated cells generally showed higher EIF4E mRNA expression than the corresponding untreated controls, with broadly increasing patterns across exposure concentrations and durations.

#### 2.8.2. Changes in NCBP1 mRNA Expression Following PFOS Exposure

In Nthy-ori 3-1 cells, mean NCBP1 mRNA expression was generally higher in the PFOS-treated groups than in the corresponding untreated controls. Expression tended to be higher after longer exposure durations, and the 200 μmol/L group generally showed the highest mean expression. The magnitude of the response nevertheless varied across individual concentrations and time points (Figure 12c).

In TPC-1 cells, mean NCBP1 mRNA expression also generally increased with exposure duration following PFOS treatment. Higher mean expression was often observed at the higher PFOS concentrations, with the 200 μmol/L group generally showing the highest values. Across the evaluated exposure groups, the highest mean expression values were observed at 144 h (Figure 12d).

Overall, PFOS-treated cells generally showed higher NCBP1 mRNA expression than the corresponding untreated controls, with broadly increasing patterns across exposure concentrations and durations. These gene-expression changes should not be interpreted as direct evidence of altered intracellular m^7^G modification levels. Two-way ANOVA showed significant effects of PFAS concentration, exposure duration, and concentration-by-time interaction on EIF4E mRNA expression following PFOA exposure and on NCBP1 mRNA expression following PFOS exposure in both Nthy-ori 3-1 and TPC-1 cells (all *p* < 0.001).

## 3. Discussion

This study integrated bioinformatics analyses, a hospital-based case–control study, and in vitro experiments to investigate the associations between per- and polyfluoroalkyl substances (PFAS) exposure and papillary thyroid carcinoma (PTC) and to explore the potential involvement of m^7^G-associated RNA regulation. Three principal findings emerged. First, integration of PFAS-associated and m^7^G-associated gene sets identified 24 overlapping candidate genes, of which 21 remained significantly differentially expressed between PTC and normal thyroid tissues after FDR correction. Network-topology analysis further identified EIF4E, NCBP1, and AGO2 as candidate hub genes. Second, several individual PFAS were inversely associated with the odds of PTC, with six associations remaining significant after FDR correction in the continuous models; qgcomp and BKMR showed broadly consistent inverse mixture patterns, whereas WQS estimates were not statistically significant. Third, exposure to high concentrations of PFOA and PFOS reduced relative thyroid-cell viability in vitro, wound-healing responses were variable without a consistent enhancement, and PFOA and PFOS exposure was accompanied by changes in EIF4E and NCBP1 mRNA expression, respectively.

Collectively, these findings provide exploratory evidence of associations among PFAS exposure, PTC, and m^7^G-associated RNA regulatory processes. However, the bioinformatics, epidemiological, and cellular components represent distinct levels of evidence obtained under different study conditions and should not be interpreted as forming a continuous causal pathway. Accordingly, the present study cannot establish a causal relationship between PFAS exposure and PTC or demonstrate that PFAS alters intracellular m^7^G modification.

### 3.1. PFAS-Associated m^7^G Candidate Genes and Post-Transcriptional RNA Regulation in PTC

Intersecting the PFAS-associated and m^7^G-associated gene sets identified 24 overlapping candidate genes, of which 21 remained significantly differentially expressed between PTC and normal thyroid tissues after false discovery rate correction. Functional enrichment analysis highlighted processes related to translation initiation, RNA localization and degradation, RNA cap binding, nucleocytoplasmic transport, and mRNA surveillance. Together, these findings support the involvement of post-transcriptional RNA regulatory processes in the molecular landscape associated with PFAS-related candidate genes in PTC [25], in addition to previously proposed processes such as endocrine disruption [37], oxidative stress, and inflammation [12,14]. PFAS exposure data were not available for the TCGA samples, so the transcriptomic patterns cannot be directly linked to individual PFAS exposure levels in that cohort.

N^7^-methylguanosine is a defining component of the eukaryotic mRNA 5′ cap and contributes to mRNA stability, nuclear export, and cap-dependent translation [27]. Alterations in cap recognition, RNA stability, and translation initiation may influence cellular stress responses and the expression of tumor-related proteins [28,29]. In the present analysis, enriched molecular-function terms included RNA cap binding and RNA 7-methylguanosine cap binding, whereas enriched pathways included RNA degradation, nucleocytoplasmic transport, and mRNA surveillance. These annotation patterns are consistent with potential roles of the candidate-gene set in RNA homeostasis and cap-associated post-transcriptional regulation. Nevertheless, enrichment analysis identifies statistical overrepresentation of annotated functions and cannot demonstrate pathway activity or changes in intracellular m^7^G modification levels.

PPI-network analysis showed that EIF4E, NCBP1, and AGO2 consistently ranked among the leading candidates according to the Degree, MCC, and MNC algorithms. EIF4E recognizes the mRNA 5′ cap and facilitates cap-dependent translation initiation. NCBP1 is a component of the nuclear cap-binding complex and participates in nascent RNA processing, transcript stability, and nuclear export. By contrast, AGO2 is not a direct mRNA cap-binding protein but is a central component of the RNA-induced silencing complex and mediates microRNA-dependent post-transcriptional gene silencing. Their recurrent identification across network-topology approaches, together with the enrichment of cap- and RNA-processing-related functions, supports their relevance as priority candidates for further investigation of PFAS-responsive RNA regulatory processes in PTC.

### 3.2. Associations of Candidate Hub-Gene Expression with Estimated Immune-Cell Proportions in PTC

Computational deconvolution analysis showed that, at the nominal significance threshold of *p* < 0.05, the estimated proportions of 16 immune-cell subsets differed between PTC and normal thyroid tissues. These differences involved several T-cell, macrophage, dendritic-cell, and other immune-cell subsets, which is consistent with previous studies reporting alterations in the immune microenvironment of PTC [38,39,40]. However, these estimates were derived from bulk transcriptomic data and should not be interpreted as direct histological measurements of immune-cell infiltration.

The expression of EIF4E, NCBP1, and AGO2 was associated with the estimated proportions of several immune-cell subsets. EIF4E and NCBP1 expression was positively correlated with the estimated proportions of resting memory CD4^+^ T cells and eosinophils and negatively correlated with those of regulatory T cells and resting dendritic cells. AGO2 expression was also correlated with the estimated proportions of several T-cell subsets, plasma cells, and natural killer cells. These findings indicate statistical associations between candidate-gene expression and computationally estimated immune composition but do not demonstrate direct regulation of immune-cell recruitment or function by these genes.

Several limitations should be considered when interpreting these findings. Computational deconvolution may not fully capture the spatial distribution, activation states, or functional heterogeneity of immune cells within tumor tissues. In addition, cross-sectional correlations cannot determine whether candidate-gene expression contributes to changes in immune-cell composition, results from tumor-associated immune alterations, or reflects another shared biological process. Individual PFAS exposure data were also unavailable for the TCGA cohort. Therefore, the current analysis cannot directly establish a pathway linking PFAS exposure, candidate-gene expression, and immune microenvironmental changes.

Overall, EIF4E, NCBP1, and AGO2 should be regarded as candidate genes associated with estimated immune microenvironmental features in PTC rather than confirmed mediators of PFAS-related immune responses. Their potential roles require further investigation using direct tissue-based and functional approaches, including immunohistochemistry, flow cytometry, single-cell RNA sequencing, spatial transcriptomics, and gene-manipulation experiments.

### 3.3. Associations of Individual Serum PFAS Concentrations with the Odds of PTC

Seventeen PFAS were measured in serum samples, and 12 compounds were retained for the principal association analyses. PFOS and PFOA were detected in all participants and had the highest median serum concentrations among the measured compounds. Positive correlations of varying strengths were also observed among several PFAS, indicating correlated exposure patterns within the study population and supporting the need to consider both individual compounds and PFAS mixtures.

After adjustment for age, sex, BMI, place of residence, smoking status, and alcohol consumption, higher serum concentrations of 9Cl-PF3ONS, PFOS, PFUnA, PFDA, PFBA, PFNA, and PFTrDA were associated with lower odds of PTC in the continuous-exposure models. After FDR correction, these associations remained significant except for PFNA. The tertile analyses also showed inverse associations for several compounds, and the trends for 9Cl-PF3ONS, PFUnA, PFBA, and PFTrDA remained statistically significant after false discovery rate correction. Restricted cubic spline analysis provided evidence of a nonlinear association for PFBA. The overall associations for 9Cl-PF3ONS, PFUnA, and PFTrDA were statistically significant, but there was no clear evidence of deviation from linearity. These findings suggest that the magnitude and shape of the associations varied across individual PFAS and analytical approaches.

Importantly, the observed inverse associations should not be interpreted as evidence that PFAS protect against PTC. This was a hospital-based case–control study, and serum samples were collected after PTC diagnosis. The temporal sequence between PFAS exposure and disease development therefore could not be established. Disease-related physiological changes, as well as changes in diet, lifestyle, thyroid function, or systemic metabolism before or around diagnosis, may have affected PFAS absorption, distribution, protein binding, and elimination. Lower serum concentrations of some PFAS among PTC cases may consequently have been influenced by reverse causation.

Serum PFAS concentrations are also affected by compound-specific and individual toxicokinetic factors, including biological half-life, serum protein binding, renal transport, and excretion [41]. Although the regression models included several major covariates, residual confounding may remain because detailed information on dietary intake, drinking-water sources, renal function, serum albumin, serum lipids, reproductive history, occupational exposure, and other environmental pollutants was unavailable or incomplete. The observed inverse associations may therefore reflect a combination of exposure patterns, disease-related changes, toxicokinetic variability, and unmeasured confounding.

Differences in carbon-chain length, functional groups, biological persistence, and tissue distribution may further contribute to the heterogeneous associations across PFAS. Shorter-chain PFAS are generally eliminated more rapidly and may more strongly reflect recent exposure, whereas longer-chain compounds tend to have greater persistence [7] and stronger serum protein binding [8]. A single serum measurement may therefore represent different exposure windows for different PFAS and may not adequately characterize long-term cumulative exposure.

The results from continuous, tertile-based, and restricted cubic spline analyses were not fully consistent. Categorization may identify differences within particular exposure ranges but results in the loss of continuous information, whereas linear models may not capture thresholds or non-monotonic patterns. Correlations among PFAS also limit the ability of single-pollutant models to separate compound-specific associations [20,21]. The findings should therefore be interpreted collectively across the different analyses rather than on the basis of isolated statistically significant estimates.

Finally, the ORs estimated in this study describe associations between serum PFAS concentrations and PTC case status within the case–control sample. They should not be interpreted as prospective estimates of PTC incidence or as evidence that increasing PFAS exposure would reduce the risk of developing PTC. Prospective studies with repeated prediagnostic exposure measurements are required to establish temporality and clarify the direction of these associations.

### 3.4. Differences in PFAS Mixture Associations Across Statistical Models

Individuals are exposed to multiple PFAS simultaneously, and several PFAS concentrations were positively correlated in the present study. These correlations complicate the estimation of compound-specific associations and may influence the results obtained from different mixture-modeling approaches.

Taken together, qgcomp and BKMR converged on a generally inverse mixture pattern, whereas the WQS estimates were not statistically significant. The differences across models likely reflect their distinct assumptions regarding exposure directionality, nonlinearity, and interactions. The consistency of the BKMR results across alternative knot specifications strengthens confidence in the overall modeled pattern, although larger prospective datasets will be important for evaluating its reproducibility.

The differences across these models may reflect their distinct assumptions and estimation procedures. Each WQS model constrains the contributions of the included mixture components to the same direction within the fitted index. Although separate positive- and negative-direction models were evaluated, WQS may have limited ability to characterize mixtures containing components with heterogeneous, nonlinear, or opposing associations. qgcomp permits individual components to contribute in both positive and negative directions and may therefore be more flexible for mixtures with heterogeneous component associations. BKMR can accommodate nonlinear exposure–response functions and potential interactions, but its estimates may be imprecise or unstable when the sample size is limited relative to the number and correlation of the exposure variables.

PFBA had a high posterior inclusion probability in the reported BKMR models, indicating relatively high variable importance within those fitted models. However, PIP is a model-dependent measure and does not represent the magnitude of a causal effect. In a correlated mixture, a high PIP may indicate that a compound carries information about its own concentration, other correlated compounds, or a broader shared exposure pattern. PFBA should therefore not be interpreted as the sole driver of the modeled mixture association, and causal priority cannot be assigned to individual PFAS solely on the basis of PIP rankings [24].

Overall, qgcomp provided statistical evidence of an inverse mixture association, and BKMR showed a generally inverse modeled pattern, whereas the WQS analyses were not statistically significant. This incomplete agreement indicates the sensitivity of mixture analyses to model assumptions and supports an exploratory interpretation of the findings. Moreover, the case–control design, limited sample size, correlated exposures, and potential reverse causation prevent the inverse estimates from being interpreted as evidence of a protective effect of PFAS mixtures. Replication in larger prospective populations is needed to determine whether these patterns are robust.

### 3.5. Cellular Responses to PFOA and PFOS and Expression of Selected Candidate Genes

The wound-healing assay showed variable and non-monotonic responses across PFOA and PFOS concentrations in both cell lines. Although several PFOS-treated TPC-1 groups showed numerically higher mean wound-closure rates at 24 h, no consistent enhancement was observed across the tested conditions. By 48 h, wounds in the control and most exposure groups were nearly closed, producing a clear ceiling effect. These findings therefore do not support a stable wound-closure-promoting effect of PFOA or PFOS under the present experimental conditions.

The CCK-8 results showed relatively limited changes in the metabolic activity of both thyroid cell lines following exposure to low-to-moderate concentrations of PFOA or PFOS. At higher concentrations, relative viability generally decreased, with more pronounced reductions observed at higher exposure levels and longer exposure durations. These findings suggest cytotoxic effects under high-concentration conditions but do not provide evidence of a persistent proliferation-promoting effect at lower concentrations.

Because the CCK-8 assay primarily reflects cellular metabolic activity, it cannot be regarded as a direct measurement of cell proliferation. The reductions in relative viability observed at high PFAS concentrations may be related to oxidative stress, mitochondrial dysfunction, endoplasmic reticulum stress, or cell death [14,42,43,44]. However, these processes were not measured directly, and their involvement cannot be determined from the present experiments.

The qRT-PCR analyses showed concentration- and time-dependent changes in EIF4E expression following PFOA exposure and in NCBP1 expression following PFOS exposure. Two-way ANOVA demonstrated significant effects of concentration, exposure duration, and their interaction in both thyroid cell lines. These results indicate that the selected candidate genes are responsive to PFAS exposure and support the involvement of cap-associated and post-transcriptional RNA regulatory processes in thyroid-cell responses.

The qRT-PCR analyses showed that PFOA-treated cells generally had higher EIF4E mRNA expression than the corresponding untreated controls, with broadly increasing patterns across concentrations and exposure durations. Similarly, PFOS-treated cells generally showed higher NCBP1 mRNA expression, although the magnitude of the response varied across concentrations and time points. These findings suggest that the selected candidate genes responded to PFAS exposure under the experimental conditions.

EIF4E participates in mRNA cap recognition and cap-dependent translation [33], whereas NCBP1 is a component of the nuclear cap-binding complex involved in RNA processing, quality control, stability, and nuclear export [34,35]. Changes in their expression may therefore be relevant to post-transcriptional RNA regulation and cellular stress responses. Nevertheless, increased EIF4E or NCBP1 mRNA expression does not demonstrate that PFOA directly alters cap-dependent translation or that PFOS directly affects nuclear cap-binding activity and RNA processing.

Importantly, altered EIF4E or NCBP1 expression does not demonstrate changes in intracellular m^7^G modification. The study did not measure EIF4E or NCBP1 protein abundance, cap-binding activity, global or transcript-specific m^7^G levels, or downstream translational activity. Functional interventions involving gene knockdown or overexpression were also not performed. The findings therefore indicate changes in selected candidate-gene expression rather than a causal pathway linking PFAS exposure, altered m^7^G modification, and thyroid-cell phenotypes.

The micromolar concentrations used in the cellular experiments were substantially higher than the nanogram-per-milliliter serum concentrations observed in the study population. These doses were selected from preliminary viability experiments to characterize measurable cellular responses while avoiding extensive cytotoxicity. Accordingly, the in vitro experiments should be viewed as response-characterization studies under controlled exposure conditions rather than direct simulations of chronic environmental exposure. Together with the epidemiological findings, they provide complementary information on population-level exposure patterns and thyroid-cell responses to PFAS.

### 3.6. Strengths, Limitations, and Future Directions

This study has several strengths. First, it integrated bioinformatics analyses, a hospital-based case–control study, and targeted in vitro experiments to investigate PFAS and PTC from complementary perspectives. These components provided information on candidate-gene screening, population-level exposure associations, and cellular responses at different levels of evidence.

Second, the epidemiological component evaluated both individual PFAS and PFAS mixtures. The combined use of logistic regression, restricted cubic splines, WQS regression, qgcomp, and BKMR enabled the assessment of continuous and categorical associations, potential nonlinear relationships, overall mixture patterns, and the relative importance of individual components under different modeling assumptions.

Third, the bioinformatics analyses identified EIF4E, NCBP1, and AGO2 as candidate hub genes based on network topology and evaluated their associations with estimated immune-cell proportions. The cellular experiments further demonstrated changes in EIF4E and NCBP1 mRNA expression following selected PFAS treatments. This convergence at the candidate-gene level supports further investigation of post-transcriptional RNA regulation but does not imply experimental validation of the epidemiological or bioinformatics findings.

Several limitations should also be acknowledged. First, the hospital-based case–control design could not establish whether PFAS exposure preceded the onset of PTC, and recruitment from a single hospital may limit the generalizability of the findings. In addition, thyroid ultrasonography and biochemical thyroid screening were not routinely performed in all controls, so occult thyroid abnormalities could not be completely excluded.

Second, the sample size was relatively limited, particularly for models involving multiple correlated PFAS and several covariates. This may have reduced statistical power, widened confidence intervals, and increased the instability of mixture-model and subgroup estimates. Furthermore, several PFAS had relatively low detection frequencies, making the corresponding estimates more sensitive to LOD substitution and the distribution of imputed values.

Third, the bioinformatics component relied on publicly available databases, predicted chemical targets, and transcriptomic data from populations without individual PFAS exposure measurements. Although differential expression findings remained significant after FDR correction, database-derived and predicted target relationships still require direct experimental validation.

The wound-healing assay also reflects multiple cellular processes, and the qRT-PCR experiments evaluated selected PFAS–gene pairs using mRNA expression as the primary molecular endpoint. These design features define the scope of the experimental findings and highlight priorities for subsequent protein-level and functional validation.

Fourth, the in vitro experiments used short-term, single-compound exposures at concentrations substantially higher than those measured in human serum. These experiments could not reproduce long-term, low-dose, or mixed human exposure conditions. In addition, the wound-healing assay could not distinguish cell migration from changes in proliferation, viability, or adhesion. Only EIF4E and NCBP1 mRNA expression was measured, whereas protein expression, m^7^G modification, translational activity, functional gene intervention, and in vivo responses were not evaluated. The qRT-PCR results also relied on normalization to a single reference gene, GAPDH, whose stability under PFAS exposure remains uncertain.

Future studies should include larger, multicenter, prospective populations with repeated prediagnostic PFAS measurements to clarify temporal relationships and exposure–response patterns. More comprehensive information on renal function, serum proteins and lipids, dietary intake, drinking-water sources, occupational factors, and co-exposure to other environmental contaminants should also be collected. Experimental studies should evaluate low-dose, long-term, and PFAS mixture exposures that more closely resemble environmental conditions. In addition, measurement of EIF4E and NCBP1 protein expression, global and transcript-specific m^7^G modifications, cap-binding activity, translational activity, and functional manipulation of candidate genes will be necessary to determine whether the observed expression changes have mechanistic relevance.

In summary, this integrated study identified PFAS-associated m^7^G candidate genes with robust differential-expression signals after FDR correction and characterized individual and mixture associations between serum PFAS concentrations and PTC case status. Several individual PFAS remained inversely associated with PTC after multiple-testing correction, while qgcomp and BKMR showed broadly consistent inverse mixture patterns. In vitro, higher concentrations of PFOA and PFOS reduced relative thyroid-cell viability, wound-healing responses remained variable, and selected PFAS exposures were accompanied by concentration- and time-dependent changes in EIF4E and NCBP1 mRNA expression. Together, these findings strengthen the rationale for further investigating PFAS-responsive post-transcriptional RNA regulation in thyroid carcinogenesis.

## 4. Materials  and Methods

### 4.1. Study Design

This study integrated bioinformatics analyses, a hospital-based case–control study, and in vitro experiments to investigate the associations between per- and polyfluoroalkyl substances (PFAS) exposure and papillary thyroid carcinoma (PTC) case status and to explore the potential involvement of m^7^G-associated RNA regulatory pathways. The study consisted of three sequential components.

First, transcriptomic data from PTC and normal thyroid tissues were obtained from The Cancer Genome Atlas Thyroid Carcinoma project (TCGA-THCA). PFAS-associated target genes were retrieved from the Comparative Toxicogenomics Database (CTD) and SwissTargetPrediction, whereas m^7^G-associated genes were collected from the Molecular Signatures Database (MSigDB) and previously published studies. These datasets were integrated to identify candidate genes at the intersection of PFAS-associated targets, PTC transcriptomic alterations, and m^7^G-associated RNA regulation. Differential expression analysis, functional enrichment analysis, protein–protein interaction (PPI) network construction, and immune-infiltration analysis were subsequently performed.

Second, a hospital-based case–control study involving 60 patients with newly diagnosed PTC and 60 frequency-matched controls was conducted. Serum concentrations of 17 PFAS were measured, and the associations between individual PFAS and PTC case status were evaluated using multivariable logistic regression and restricted cubic spline analyses. Weighted quantile sum (WQS) regression, quantile g-computation (qgcomp), and Bayesian kernel machine regression (BKMR) were used to evaluate associations between PFAS mixtures and PTC case status.

Finally, based on the bioinformatics findings and the exposure profiles observed in the study population, perfluorooctanoic acid (PFOA) and perfluorooctane sulfonate (PFOS) were selected for the in vitro experiments. Bioinformatics analyses identified EIF4E, NCBP1, and AGO2 as candidate hub genes. Because the experimental component was designed for the targeted evaluation of selected candidate genes rather than a comprehensive assessment of all bioinformatics-derived candidates, EIF4E and NCBP1 were selected for further evaluation based on their established roles in mRNA cap recognition and cap-associated translation or RNA processing, together with the feasibility of the planned cellular assays. AGO2, which primarily participates in microRNA-mediated gene silencing as a component of the RNA-induced silencing complex, was retained as a bioinformatics-derived candidate but was not included in the in vitro experiments. Human normal thyroid epithelial cells (Nthy-ori 3-1) and PTC-derived TPC-1 cells were exposed to PFOA or PFOS to evaluate cell viability, wound closure, and EIF4E and NCBP1 mRNA expression.

### 4.2. Bioinformatics Analysis

RNA-sequencing data from PTC and normal thyroid tissues were obtained from the TCGA Thyroid Carcinoma project (TCGA-THCA) [45]. PFAS-associated genes were obtained from two complementary sources. Reported chemical–gene associations were retrieved from the Comparative Toxicogenomics Database (CTD) [46], whereas predicted molecular targets were obtained from SwissTargetPrediction [47]. Each of the 17 PFAS measured in the epidemiological study was queried individually, and relevant gene information was available for 14 compounds. The retrieved genes were standardized to official gene symbols, merged after retaining their source information, and deduplicated, yielding 955 unique PFAS-associated genes. The source categories used in candidate-gene screening are summarized in Table S13.

The m^7^G-associated gene set was compiled from the Molecular Signatures Database (MSigDB) and previously published studies. After standardization to official gene symbols and removal of duplicates, 43 unique m^7^G-associated genes were retained. Overlapping genes were identified by intersecting the PFAS-associated gene set with the m^7^G-associated gene set and were defined as PFAS-associated m^7^G candidate genes.

Bioinformatics analyses were performed using R software (version 4.5.2; R Core Team, Vienna, Austria). Differential expression analysis of the overlapping candidate genes between PTC and normal thyroid tissues was performed using the limma package [48]. *p* values from the differential-expression analysis of the 24 candidate genes were adjusted for multiple testing using the Benjamini–Hochberg false discovery rate method, with FDR < 0.05 considered statistically significant. Functional enrichment analyses of the differentially expressed candidate genes were performed using the clusterProfiler package [49], including Gene Ontology (GO) and Kyoto Encyclopedia of Genes and Genomes (KEGG) pathway analyses.

All 24 overlapping candidate genes were submitted to the STRING database to construct an initial protein–protein interaction network [50]. A second PPI network was constructed using the differentially expressed candidate genes and visualized using Cytoscape software 3.10.1 [51]. Candidate hub genes were identified using the cytoHubba plugin [52] and three topological algorithms: Degree, Maximal Clique Centrality (MCC), and Maximum Neighborhood Component (MNC). Genes consistently ranked among the leading candidates across the three algorithms were retained as candidate hub genes.

Among the candidate hub genes identified, EIF4E and NCBP1 were selected for targeted experimental evaluation because of their established roles in mRNA cap recognition and cap-associated translation or RNA processing, together with the feasibility of quantitative expression analysis in the established cell models. AGO2 was retained as a bioinformatics-derived candidate but was not included in the in vitro experiments because its principal function is related to microRNA-mediated post-transcriptional silencing rather than direct mRNA cap recognition.

The ESTIMATE algorithm [53] was used to calculate stromal, immune, and ESTIMATE scores in PTC and normal thyroid tissues. The CIBERSORT algorithm [54] was used to estimate the relative proportions of 22 immune-cell subsets. Spearman correlation analysis was subsequently performed to evaluate associations between candidate hub-gene expression and the estimated immune-cell proportions.

### 4.3. Hospital-Based Case–Control Study

A hospital-based case–control study was conducted between July and October 2025 at an affiliated hospital of Xinjiang Medical University. Patients with newly diagnosed, histopathologically confirmed PTC who had not received anticancer treatment before enrollment were recruited as cases. Individuals undergoing routine health examinations at the same hospital during the same recruitment period were recruited as controls. Controls were frequency matched to cases by sex and age within ±3 years and were enrolled under the same questionnaire assessment and fasting blood-sampling protocol. A total of 120 participants were enrolled, including 60 PTC cases and 60 controls.

Demographic and lifestyle information, including age, sex, body mass index (BMI), place of residence, smoking status, and alcohol consumption, was collected using standardized questionnaires and medical-record review. Fasting venous blood samples were collected from all participants for the measurement of serum PFAS concentrations.

The study protocol was approved by the Ethics Committee of Xinjiang Medical University (approval No. XJYKDXR20250701001). Written informed consent was obtained from all participants before enrollment.

### 4.4. Determination of Serum PFAS Concentrations

Fasting venous blood samples were collected from all participants. Serum was separated and stored at −80 °C until analysis. Serum concentrations of 17 PFAS were measured, including perfluorobutanoic acid (PFBA), perfluoropentanoic acid (PFPeA), perfluorobutane sulfonate (PFBS), perfluoropentane sulfonate (PFPeS), perfluoroheptanoic acid (PFHpA), perfluorohexane sulfonate (PFHxS), perfluorooctanoic acid (PFOA), perfluoroheptane sulfonate (PFHpS), perfluorononanoic acid (PFNA), perfluorooctane sulfonate (PFOS), perfluorodecanoic acid (PFDA), perfluoroundecanoic acid (PFUnA), 9Cl-PF3ONS, perfluorododecanoic acid (PFDoA), 11Cl-PF3OUdS, perfluorotridecanoic acid (PFTrDA), and perfluorotetradecanoic acid (PFTeDA).

Serum PFAS concentrations were quantified using ultra-high-performance liquid chromatography coupled with tandem mass spectrometry (UHPLC–MS/MS) and isotope-labeled internal standards. All analytes were measured in negative electrospray ionization mode using multiple reaction monitoring (MRM).

Analytical quality-control procedures included procedural blanks, mobile-phase blanks, and matrix blanks. Background concentrations were corrected using procedural blanks. Calibration curves had coefficients of determination greater than 0.996. Analytical recoveries and precision were evaluated according to prespecified quality-control criteria. Compound-specific precursor and product ions, limits of detection and quantification, calibration ranges, recoveries, and relative standard deviations are presented in Tables S8 and S9.

Concentrations below the limit of detection (LOD) were replaced with LOD/√2 before data transformation and statistical analysis [55]. Because serum PFAS concentrations were right-skewed, the concentrations were natural logarithm-transformed to reduce skewness and improve numerical stability. Based on the prespecified analytical inclusion criteria, 12 PFAS were retained for the principal association analyses: PFBA, PFHpA, PFHxS, PFOA, PFNA, PFOS, PFDA, PFUnA, 9Cl-PF3ONS, PFDoA, PFTrDA, and PFTeDA.

### 4.5. Cell Culture and PFAS Exposure

Human normal thyroid epithelial cells (Nthy-ori 3-1) and PTC-derived TPC-1 cells were cultured in RPMI-1640 medium supplemented with 10% fetal bovine serum (FBS) and 1% penicillin–streptomycin at 37 °C in a humidified atmosphere containing 5% CO_2_.

Because PFOA and PFOS exhibited different cytotoxicity profiles in the preliminary dose-finding experiments, exposure concentrations were selected separately for each compound based on the CCK-8 results. Concentrations associated with limited to moderate reductions in relative viability were selected to permit evaluation of cellular responses while avoiding conditions associated with extensive cytotoxicity. For the wound-healing assay, cells were exposed to PFOA at concentrations of 0, 100, 150, 175, 200, or 250 μmol/L or to PFOS at concentrations of 0, 50, 100, 150, 175, or 200 μmol/L. For the quantitative real-time PCR analyses, cells were exposed to PFOA or PFOS at concentrations of 0, 100, 150, or 200 μmol/L.

### 4.6. Cell Viability Assay

Cellular metabolic activity, used as an indicator of relative cell viability, was assessed using the Cell Counting Kit-8 (CCK-8) assay. Nthy-ori 3-1 and TPC-1 cells in the logarithmic growth phase were seeded in 96-well plates at a density of 1 × 10^4^ cells per well and allowed to adhere for 12 h. The cells were then exposed to PFOA or PFOS at final concentrations of 0, 50, 100, 150, 200, 250, 300, or 500 μmol/L for 48, 96, or 144 h.

After exposure, 10 μL of CCK-8 reagent was added to each well, followed by incubation for 2 h. Absorbance was measured at 450 nm using a microplate reader. After subtraction of the blank-control absorbance, relative cell viability was expressed as a percentage of the corresponding untreated control. Each treatment condition included six technical replicates within each experiment, and the assay was performed in three independent experiments.

### 4.7. Wound-Healing Assay

Cell wound closure was assessed using a wound-healing assay. Nthy-ori 3-1 and TPC-1 cells in the logarithmic growth phase were seeded in culture plates and grown to approximately 90% confluence. A linear scratch was created across the cell monolayer using a sterile pipette tip. Detached cells were removed by washing with phosphate-buffered saline (PBS), and fresh culture medium containing PFOA or PFOS at the concentrations specified in Section 4.5 was added.

Images of the same predefined microscopic fields were acquired at 0, 24, and 48 h after treatment. Wound areas were quantified using ImageJ software 1.54f [56]. The wound-closure rate was calculated as [(A_0_ − A_t_)/A_0_] × 100%, where A_0_ and A_t_ represent the wound areas at 0 h and at each subsequent time point, respectively. The assay was performed in three independent experiments.

### 4.8. Quantitative Real-Time PCR

Following exposure to PFOA or PFOS at the concentrations specified in Section 4.5, Nthy-ori 3-1 and TPC-1 cells were harvested at 24, 48, 72, 96, 120, and 144 h. EIF4E mRNA expression was evaluated following PFOA exposure, whereas NCBP1 mRNA expression was evaluated following PFOS exposure. These representative compound–gene pairs were selected for targeted expression analysis based on the bioinformatics findings and the planned experimental design. Total RNA was extracted according to the manufacturer’s instructions and reverse-transcribed into complementary DNA (cDNA). Quantitative real-time PCR (qRT-PCR) was performed using SYBR Green chemistry, with GAPDH serving as the internal reference gene.

Relative EIF4E and NCBP1 mRNA expression was normalized to GAPDH and calculated using the 2^−^^ΔΔCt^  method [57], with untreated Nthy-ori 3-1 cells used as the calibrator. Each reaction was performed in technical triplicate, and the experiments were independently repeated at least three times. The primer sequences are provided in Table S10.

### 4.9. Statistical Analysis

All statistical analyses were performed using SPSS software (version 26.0; IBM Corp., Armonk, NY, USA) and R software (version 4.5.2). Continuous variables were summarized as the mean ± standard deviation (SD) or the median and interquartile range (IQR), as appropriate, whereas categorical variables were summarized as frequencies and percentages. Between-group comparisons of continuous variables were performed using the independent-samples *t* test or the Mann–Whitney U test, as appropriate. Categorical variables were compared using the chi-square test or Fisher’s exact test. Serum PFAS concentrations were compared between PTC cases and controls using the Mann–Whitney U test. Spearman correlation coefficients were calculated to characterize correlations among serum PFAS concentrations.

Serum PFAS concentrations below the LOD were imputed as LOD/√2, after which the PFAS concentrations were natural logarithm-transformed. Associations between individual PFAS concentrations and PTC case status were evaluated using multivariable logistic regression. PFAS concentrations were analyzed as continuous ln-transformed variables and as tertiles defined by the 33rd and 66th percentiles, with the lowest tertile serving as the reference category. Odds ratios (ORs) and 95% confidence intervals (CIs) were estimated after adjustment for age, sex, body mass index, place of residence, smoking status, and alcohol consumption. Tests for trend were conducted across the tertile categories. The same tertile-based strategy was applied in the sex- and BMI-stratified analyses. To account for multiple comparisons in the continuous single-PFAS models, *p* values across the 12 PFAS were adjusted using the Benjamini–Hochberg false discovery rate procedure. Given the fixed sample size of 60 PTC cases and 60 controls, an approximate minimum detectable effect analysis was used to characterize the statistical resolution of the epidemiological analyses. Under a two-sided α of 0.05 and 80% power, the overall sample was estimated to detect odds ratios of approximately 0.60 or 1.67 per standard-deviation increase in a continuous exposure under an idealized balanced case–control setting. Subgroup findings were treated as secondary analyses because of their smaller sample sizes.

Restricted cubic spline (RCS) analyses were performed using the ln-transformed PFAS concentrations to evaluate potential nonlinear exposure–response relationships [58]. Associations between PFAS mixtures and PTC case status were evaluated using weighted quantile sum regression [22], quantile g-computation [23], and Bayesian kernel machine regression (BKMR) based on the ln-transformed PFAS concentrations [24]. WQS regression was fitted separately under positive- and negative-direction constraints, with component weights used to characterize the relative contribution of individual PFAS to the mixture index. For quantile g-computation, PFAS concentrations were categorized into quartiles, and the overall mixture effect was estimated for a joint one-quartile increase in all mixture components; positive and negative component weights were calculated to characterize the relative contribution and direction of individual PFAS. Before BKMR model fitting, ln-transformed PFAS concentrations were standardized to a mean of 0 and a standard deviation of 1. BKMR models were run for 20,000 Markov chain Monte Carlo iterations. The primary analysis used 10 knots, and model convergence and mixing were assessed using trace plots and MCMC acceptance diagnostics. A sensitivity analysis using 50 knots was performed to evaluate the stability of the estimated overall mixture association and posterior inclusion probabilities.

For the cell experiments, data are presented as the mean ± SD. Technical replicates were averaged within each independent experiment, and the independent experiments were treated as the analytical units. For qRT-PCR experiments involving multiple PFAS concentrations and exposure durations, two-way ANOVA was used to evaluate the main effects of concentration and time and their interaction. Where appropriate, post hoc comparisons were performed against the corresponding untreated control. Other cell-experiment comparisons were analyzed according to the experimental design using independent-samples *t* tests or one-way ANOVA. All statistical tests were two-sided, and *p* < 0.05 was considered statistically significant.

## 5. Conclusions

This study integrated bioinformatics analyses, a hospital-based case–control study, and in vitro experiments to investigate associations between PFAS exposure and papillary thyroid carcinoma (PTC) at the molecular, epidemiological, and cellular levels. Bioinformatics analyses identified 24 PFAS-associated m^7^G candidate genes, of which 21 remained significantly differentially expressed between PTC and normal thyroid tissues after false discovery rate correction. EIF4E, NCBP1, and AGO2 were consistently identified as candidate hub genes involved in post-transcriptional RNA regulation, with EIF4E and NCBP1 further selected for targeted experimental evaluation.

The case–control study identified inverse associations between several individual PFAS and PTC case status, with 9Cl-PF3ONS, PFOS, PFUnA, PFDA, PFBA, and PFTrDA remaining statistically significant after FDR correction in the continuous models. qgcomp and BKMR showed broadly consistent inverse mixture patterns, and the principal BKMR findings remained stable under alternative knot specifications, whereas the WQS estimates were not statistically significant. In vitro, higher concentrations of PFOA and PFOS reduced relative thyroid-cell viability, wound-healing responses were variable without a consistent enhancement, and selected PFAS exposures were accompanied by concentration- and time-dependent changes in EIF4E and NCBP1 mRNA expression.

Taken together, these findings characterize PFAS–PTC associations and identify m^7^G-associated post-transcriptional regulatory candidates that may contribute to thyroid cellular responses to PFAS. The integration of population-level exposure data with molecular and cellular evidence provides a basis for further investigation of PFAS-responsive RNA regulatory mechanisms and their relevance to thyroid carcinogenesis.

## Figures and Tables

**Table 1:** Characteristics of PTC cases and controls.

	All	Control	PTC	*p*
	(n = 120)	(n = 60)	(n = 60)	
Age, years-Mean (SD)	45.19 (9.9)	46.1 (9.5)	44.3 (10.3)	0.356
Sex, n (%)				1.000
Female	84 (70.0%)	42 (70.0%)	42 (70.0%)	
Male	36 (30.0%)	18 (30.0%)	18 (30.0%)	
Marital status, n (%)				
Married	108 (90.0%)	53 (88.3%)	55 (91.7%)	0.543
Others	12 (10.0%)	7 (11.7%)	5 (8.3%)	
BMI (kg/m^2^)	24.45 (3.0)	23.1 (2.2)	25.8 (3.1)	<0.001
Residence, n (%)				0.008
Urban area	99 (82.5%)	55 (91.7%)	44 (73.3%)	
Rural area	21 (17.5%)	5 (8.3%)	16 (26.7%)	
Smoking status, n (%)				0.752
Never smoked/Former smoker	109 (90.8%)	54 (90.0%)	55 (91.7%)	
Current smoker	11 (9.2%)	6 (10.0%)	5 (8.3%)	
Alcohol drinking status, (n%)				0.697
Never drank/Former drinker	113 (94.2%)	57 (95.0%)	56 (93.3%)	
Current drinker	7 (5.8%)	3 (5.0%)	4 (6.7%)	

**Table 2:** Detection frequencies and serum concentrations of PFAS in PTC cases and controls.

PFAS	LOD (ng/mL)	ALL (n = 120)		Control (n = 60)		PTC (n = 60)		*p*
		(%)	P50 (P25, P75)	(%)	P50 (P25, P75)	(%)	P50 (P25, P75)	
9Cl-PF3ONS	0.02	91.7	0.20 (0.09, 0.35)	96.7	0.22 (0.15, 0.39)	88.3	0.15 (0.06, 0.29)	0.004
PFBS	0.02	21.7	0.02 (0.02, 0.02)	41.7	0.02 (0.02, 0.03)	1.7	0.02 (0.02, 0.02)	<0.001
PFHxS	0.10	26.7	0.07 (0.05, 0.12)	31.7	0.07 (0.05, 0.14)	23.3	0.07 (0.04, 0.09)	0.217
PFOS	0.01	100.0	1.11 (0.61, 1.76)	100.0	1.21 (0.69, 1.82)	100.0	0.93 (0.51, 1.54)	0.067
PFUnA	0.01	98.3	0.21 (0.08, 0.36)	100.0	0.26 (0.13, 0.40)	98.3	0.13 (0.06, 0.30)	0.002
PFDA	0.10	68.3	0.16 (0.09, 0.25)	73.3	0.20 (0.09, 0.32)	65.0	0.15 (0.07, 0.22)	0.034
PFBA	0.10	35.0	0.08 (0.07, 0.12)	50.0	0.10 (0.08, 0.14)	20.0	0.07 (0.06, 0.09)	<0.001
PFDoA	0.01	54.2	0.01 (0.01, 0.02)	66.7	0.01 (0.01, 0.02)	43.3	0.01 (0.01, 0.02)	0.052
PFHpA	0.01	57.5	0.01 (0.01, 0.02)	63.3	0.01 (0.01, 0.02)	53.3	0.01 (0.01, 0.02)	0.407
PFNA	0.10	65.0	0.13 (0.06, 0.21)	71.7	0.15 (0.08, 0.21)	60.0	0.12 (0.05, 0.20)	0.111
PFOA	0.05	100.0	0.90 (0.55, 1.36)	100.0	0.94 (0.66, 1.37)	100.0	0.82 (0.41, 1.30)	0.102
PFPeA	0.01	100.0	0.28 (0.17, 0.28)	100.0	0.28 (0.28, 0.28)	100.0	0.18 (0.17, 0.28)	0.004
PFTeDA	0.01	100.0	0.16 (0.16, 0.17)	100.0	0.16 (0.16, 0.17)	100.0	0.16 (0.16, 0.17)	0.069
PFTrDA	0.01	56.7	0.02 (0.01, 0.04)	73.3	0.03 (0.01, 0.04)	41.7	0.01 (0.01, 0.03)	0.001

**Table 3:** Associations of ln-transformed serum PFAS concentrations with the odds of papillary thyroid carcinoma.

PFAS	Model 1 OR (95% CI)	*p*-Value	Model 2 OR (95% CI)	*p*-Value
9Cl-PF3ONS	0.59 (0.42–0.83)	0.003	0.48 (0.32–0.74)	0.001
PFHxS	0.71 (0.47–1.08)	0.113	0.74 (0.44–1.25)	0.260
PFOS	0.60 (0.38–0.96)	0.033	0.43 (0.23–0.79)	0.007
PFUnA	0.52 (0.35–0.78)	0.001	0.42 (0.25–0.69)	0.001
PFDA	0.61 (0.40–0.93)	0.022	0.53 (0.31–0.90)	0.019
PFBA	0.07 (0.02–0.25)	<0.001	0.06 (0.01–0.26)	<0.001
PFDoA	0.79 (0.58–1.08)	0.142	0.76 (0.54–1.06)	0.101
PFHpA	0.82 (0.57–1.17)	0.266	0.65 (0.42–1.03)	0.066
PFNA	0.67 (0.45–1.01)	0.053	0.60 (0.37–0.98)	0.043
PFOA	0.64 (0.40–1.02)	0.062	0.63 (0.37–1.09)	0.101
PFTeDA	0.00 (0.00–793.91)	0.125	0.00 (0.00–238,777)	0.181
PFTrDA	0.58 (0.40–0.84)	0.004	0.55 (0.34–0.90)	0.016

## Data Availability

The clinical datasets generated and analyzed during the current study are available from the corresponding author upon reasonable request. The clinical data are not publicly available due to privacy and ethical restrictions. Publicly available transcriptomic data used in this study were obtained from The Cancer Genome Atlas (TCGA) database.

## Supplementary Materials

The following supporting information can be downloaded at: https://www.mdpi.com/article/10.3390/ijms27188296/s1.

## Abbreviations

The following abbreviations are used in this manuscript:

PFAS	Per- and polyfluoroalkyl substances
PTC	Papillary thyroid carcinoma
PFOA	Perfluorooctanoic acid
PFOS	Perfluorooctane sulfonate
PFNA	Perfluorononanoic acid
PFDA	Perfluorodecanoic acid
PFUnA	Perfluoroundecanoic acid
PFDoA	Perfluorododecanoic acid
PFTrDA	Perfluorotridecanoic acid
PFTeDA	Perfluorotetradecanoic acid
PFHxS	Perfluorohexane sulfonate
PFHpA	Perfluoroheptanoic acid
PFPeA	Perfluoropentanoic acid
PFBA	Perfluorobutanoic acid
PFBS	Perfluorobutane sulfonate
LOD	Limit of detection
BMI	Body mass index
OR	Odds ratio
CI	Confidence interval
RCS	Restricted cubic spline
WQS	Weighted quantile sum
BKMR	Bayesian kernel machine regression
PIP	Posterior inclusion probability
m^7^G	N^7^-methylguanosine
