# Supplementary material for: Per- and Polyfluoroalkyl Substances and Papillary Thyroid Carcinoma: An Integrative Study of Bioinformatics, Epidemiological Associations, and In Vitro Responses
Source: Int J Mol Sci. 2026 Sep 17;27(18):8296. doi: 10.3390/ijms27188296 (PMC13607646; doi:10.3390/ijms27188296)
Supplement: Supplementary file 1 [file ijms-27-08296-s001.zip › ijms-4517194-Supplementary Figure Captions.pdf]

Figure S1. Chromosomal localization of 21 PFAS-associated m^7G candidate genes.

Figure S2. Restricted cubic spline plots showing the associations between serum PFTeDA, 9Cl-PF3ONS, PFNA, PFHpA and the odds of PTC.

Figure S3. Restricted cubic spline plots showing the associations between serum PFDoA, PFHxS, PFDA, PFTrDA and the odds of PTC.

Figure S4. Weighted quantile sum regression analysis showing the estimated weights of individual PFAS.

Figure S5. Weighted quantile sum regression analysis showing positive- and negative-direction weights for individual PFAS.

Figure S6. Quantile-specific odds ratios for PFAS mixture associated with PTC risk.

Figure S7. Quantile-specific estimates of the association between PFAS mixture and PTC risk.

Figure S8. Wound-healing assay showing wound-closure responses of Nthy-ori 3-1 cells under PFOA treatment.

Figure S9. Wound-healing assay showing wound-closure responses of Nthy-ori 3-1 cells under PFOS treatment.
