# Supplementary material for: Per- and Polyfluoroalkyl Substances and Papillary Thyroid Carcinoma: An Integrative Study of Bioinformatics, Epidemiological Associations, and In Vitro Responses
Source: Int J Mol Sci. 2026 Sep 17;27(18):8296. doi: 10.3390/ijms27188296 (PMC13607646; doi:10.3390/ijms27188296)
Supplement: Supplementary file 1 [file ijms-27-08296-s001.zip › Table S1.pdf]

Table S1. Distribution of serum PFAS concentrations among female participants

PFAS	LOD (ng/mL)	ALL (n =84)		Control (n = 42)		PTC(n = 42)		*P*
		Detection rate (%)	P_{50} (P_{25}, P_{75})	Detection rate (%)	P_{50} (P_{25}, P_{75})	Detection rate (%)	P_{50} (P_{25}, P_{75})	
9Cl-PF3ONS	0.02	92.9	0.20 (0.11, 0.34)	97.6	0.22 (0.14, 0.39)	88.1	0.19 (0.07, 0.24)	**0.043**
PFBS	0.02	23.8	0.02 (0.02, 0.02)	45.2	0.02 (0.02, 0.03)	2.4	0.02 (0.02, 0.02)	**<0.001**
PFHxS	0.10	28.6	0.07 (0.05, 0.12)	35.7	0.09 (0.05, 0.14)	21.4	0.07 (0.04, 0.09)	0.128
PFOS	0.01	100.0	1.14 (0.63, 1.79)	100.0	1.23 (0.64, 1.66)	100.0	1.01 (0.60, 1.91)	0.513
PFUnA	0.01	98.8	0.20 (0.09, 0.33)	100.0	0.23 (0.12, 0.35)	97.6	0.19 (0.07, 0.32)	0.181
PFDA	0.10	73.8	0.17 (0.09, 0.24)	73.8	0.17 (0.09, 0.25)	73.8	0.17 (0.09, 0.23)	0.598
PFBA	0.10	38.1	0.09 (0.07, 0.12)	52.4	0.11 (0.09, 0.14)	23.8	0.07 (0.06, 0.09)	**<0.001**
PFDoA	0.01	53.6	0.01 (0.01, 0.02)	59.5	0.01 (0.01, 0.02)	47.6	0.01 (0.01, 0.02)	0.593
PFHpA	0.01	59.5	0.01 (0.01, 0.03)	59.5	0.01 (0.01, 0.03)	59.5	0.01 (0.01, 0.02)	0.911
PFNA	0.10	70.2	0.13 (0.07, 0.20)	71.4	0.13 (0.07, 0.20)	69.0	0.13 (0.06, 0.20)	0.741
PFOA	0.05	100.0	0.87 (0.59, 1.37)	100.0	0.90 (0.64, 1.39)	100.0	0.87 (0.46, 1.24)	0.383
PFPeA	0.01	100.0	0.28 (0.17, 0.28)	100.0	0.28 (0.28, 0.28)	100.0	0.24 (0.17, 0.28)	**0.022**
PFTeDA	0.01	100.0	0.16 (0.16, 0.17)	100.0	0.16(0.16, 0.17)	100.0	0.16 (0.16, 0.17)	0.616
PFTrDA	0.01	57.1	0.02 (0.01, 0.03)	66.7	0.02 (0.01, 0.03)	47.6	0.02 (0.01, 0.03)	**0.172**

Note: PFAS concentrations are presented as detection rates (%) and median (P_{25}, P_{75}). Values below the limit of detection (LOD) were imputed as LOD/√2. P values were calculated using the Mann–Whitney U test. Bold values indicate statistical significance ($P < 0.05$). PTC, papillary thyroid carcinoma; PFAS, per- and polyfluoroalkyl substances.
