# Supplementary material for: Per- and Polyfluoroalkyl Substances and Papillary Thyroid Carcinoma: An Integrative Study of Bioinformatics, Epidemiological Associations, and In Vitro Responses
Source: Int J Mol Sci. 2026 Sep 17;27(18):8296. doi: 10.3390/ijms27188296 (PMC13607646; doi:10.3390/ijms27188296)
Supplement: Supplementary file 1 [file ijms-27-08296-s001.zip › Table S10.pdf]

Table S10 Primer sequences used for quantitative real-time PCR analysis

Gene	Forward primer (5′–3′)	Reverse primer (5′–3′)
EIF4E	CTGCGGCTGATCCAAG	TTCCCACATAGGCTCAATACC
NCBP1	GCTAAAGAGAAACTTGCTAGGC	GGTCAAGATCATGATAAACCGC
GAPDH	CAAGTTCAACGGCACAGTCAAGG	CGACATACTCAGCACCAGCATCA
