# Supplementary material for: Per- and Polyfluoroalkyl Substances and Papillary Thyroid Carcinoma: An Integrative Study of Bioinformatics, Epidemiological Associations, and In Vitro Responses
Source: Int J Mol Sci. 2026 Sep 17;27(18):8296. doi: 10.3390/ijms27188296 (PMC13607646; doi:10.3390/ijms27188296)
Supplement: Supplementary file 1 [file ijms-27-08296-s001.zip › Table S11.pdf]

Table S11. Estimated effects and standard deviations of PFAS mixture exposure on papillary thyroid carcinoma risk based on the Bayesian kernel machine regression (BKMR) model

Percentile	Overall population[a]				BMI subgroup[b]				Female subgroup[c]			
	knots10		knots50		Normal weight		Other		knots10		knots50	
	estimates	SD	estimates	SD	estimates	SD	estimates	SD	estimates	SD	estimates	SD
10th	1.46	0.38	1.53	0.42	4.94	1.09	0.76	0.81	1.39	0.51	1.92	0.66
18th	1.00	0.22	1.05	0.24	3.93	0.62	0.68	0.42	0.90	0.30	1.30	0.37
26th	0.73	0.15	0.77	0.17	2.43	0.45	0.38	0.29	0.62	0.20	0.90	0.24
34th	0.53	0.12	0.58	0.13	1.49	0.33	0.21	0.22	0.38	0.14	0.57	0.16
42nd	0.20	0.08	0.23	0.09	0.83	0.21	0.15	0.12	0.11	0.08	0.16	0.09
50th	0.00	0.00	0.00	0.00	0.00	0.00	0.00	0.00	0.00	0.00	0.00	0.00
58th	-0.28	0.05	-0.31	0.06	-0.63	0.20	-0.54	0.18	-0.20	0.06	-0.29	0.08
66th	-0.56	0.09	-0.60	0.10	-1.29	0.43	-0.91	0.27	-0.48	0.13	-0.65	0.17
74th	-0.87	0.14	-0.90	0.16	-1.44	0.59	-1.12	0.34	-0.71	0.19	-0.88	0.26
82nd	-1.06	0.21	-1.06	0.23	-1.43	0.68	-1.16	0.45	-0.81	0.27	-0.92	0.35
90th	-1.29	0.29	-1.24	0.32	-1.28	0.97	-1.24	0.57	-0.92	0.41	-0.85	0.49

Note: BKMR, Bayesian kernel machine regression. Effect estimates represent changes in papillary thyroid carcinoma risk associated with PFAS mixture exposure at different percentiles compared with the median exposure level (50th percentile). Covariates included age, sex, BMI, residence, smoking status, and alcohol consumption; BMI-stratified and female subgroup analyses were adjusted accordingly.
