# Supplementary material for: Per- and Polyfluoroalkyl Substances and Papillary Thyroid Carcinoma: An Integrative Study of Bioinformatics, Epidemiological Associations, and In Vitro Responses
Source: Int J Mol Sci. 2026 Sep 17;27(18):8296. doi: 10.3390/ijms27188296 (PMC13607646; doi:10.3390/ijms27188296)
Supplement: Supplementary file 1 [file ijms-27-08296-s001.zip › Table S12.pdf]

**Table S12. Posterior inclusion probabilities (PIPs) of PFAS in the Bayesian kernel machine regression (BKMR) model for the overall population and subgroups**

| PFAS | Overall population[a] | | BMI subgroup[b] | | Female(PIP)[c] | |
|---|---|---|---|---|---|---|
| | knots10 | knots50 | Normal weight | Other | knots10 | knots50 |
| 9ClPF3ONS | 0.617 | 0.752 | 0.967 | 0.390 | 0.730 | 0.821 |
| PFHxS | 0.092 | 0.188 | 0.470 | 0.402 | 0.222 | 0.440 |
| PFOS | 0.313 | 0.366 | 0.531 | 0.488 | 0.282 | 0.442 |
| PFUnA | 0.559 | 0.520 | 0.426 | 0.467 | 0.220 | 0.390 |
| PFDA | 0.229 | 0.363 | 0.590 | 0.403 | 0.212 | 0.413 |
| PFBA | 0.994 | 0.990 | 1.000 | 0.665 | 0.910 | 0.915 |
| PFDoA | 0.095 | 0.160 | 0.274 | 0.382 | 0.154 | 0.277 |
| PFHpA | 0.119 | 0.357 | 0.413 | 0.401 | 0.188 | 0.361 |
| PFNA | 0.215 | 0.312 | 0.544 | 0.380 | 0.248 | 0.467 |
| PFOA | 0.470 | 0.557 | 0.610 | 0.438 | 0.263 | 0.395 |
| PFTeDA | 0.398 | 0.444 | 0.573 | 0.431 | 0.334 | 0.467 |
| PFTrDA | 0.230 | 0.326 | 0.502 | 0.684 | 0.254 | 0.405 |

**Note:** BKMR, Bayesian kernel machine regression; PIP, posterior inclusion probability. Higher PIP values indicate greater relative importance of individual PFAS in the mixture model. Covariate adjustments were consistent with Table S8.
