# Supplementary material for: Per- and Polyfluoroalkyl Substances and Papillary Thyroid Carcinoma: An Integrative Study of Bioinformatics, Epidemiological Associations, and In Vitro Responses
Source: Int J Mol Sci. 2026 Sep 17;27(18):8296. doi: 10.3390/ijms27188296 (PMC13607646; doi:10.3390/ijms27188296)
Supplement: Supplementary file 1 [file ijms-27-08296-s001.zip › Table S13.pdf]

Table S13. Source and evidence category of PFAS-associated m^7G candidate genes

Candidate gene	Associated PFAS information	Evidence source	Evidence category
EIF4E	PFAS-associated compounds identified during candidate-gene screening	Comparative Toxicogenomics Database (CTD)	Reported chemical–gene association based on curated experimental and literature evidence
EIF4E	PFAS-associated compounds identified during candidate-gene screening	SwissTargetPrediction	Predicted molecular target based on chemical structure similarity and target prediction
NCBP1	PFAS-associated compounds identified during candidate-gene screening	Comparative Toxicogenomics Database (CTD)	Reported chemical–gene association based on curated experimental and literature evidence
NCBP1	PFAS-associated compounds identified during candidate-gene screening	SwissTargetPrediction	Predicted molecular target based on chemical structure similarity and target prediction
AGO2	PFAS-associated compounds identified during candidate-gene screening	Comparative Toxicogenomics Database (CTD)	Reported chemical–gene association based on curated experimental and literature evidence
AGO2	PFAS-associated compounds identified during candidate-gene screening	SwissTargetPrediction	Predicted molecular target based on chemical structure similarity and target prediction

Abbreviations: PFAS, per- and polyfluoroalkyl substances; CTD, Comparative Toxicogenomics Database.
