# Supplementary material for: Per- and Polyfluoroalkyl Substances and Papillary Thyroid Carcinoma: An Integrative Study of Bioinformatics, Epidemiological Associations, and In Vitro Responses
Source: Int J Mol Sci. 2026 Sep 17;27(18):8296. doi: 10.3390/ijms27188296 (PMC13607646; doi:10.3390/ijms27188296)
Supplement: Supplementary file 1 [file ijms-27-08296-s001.zip › Table S14.pdf]

Table S14 Nominal and Benjamini–Hochberg adjusted P values for differential expression of the 24 PFAS associated m^7G candidate genes

Candidate gene	Nominal P value	BH-adjusted P value	Significant after BH correction
SNUPN	0.001118	0.001578	Yes
RNMT	0.016743	0.020091	Yes
NUDT7	7.39393×10^{-20}	8.87272×10^{-19}	Yes
NUDT5	0.107492	0.117264	No
NUDT4	8.01637×10^{-4}	0.001202	Yes
NUDT16L1	3.12605×10^{-7}	9.37816×10^{-7}	Yes
NUDT16	5.52317×10^{-21}	1.32556×10^{-19}	Yes
NUDT11	7.69732×10^{-5}	1.53946×10^{-4}	Yes
NUDT10	3.14581×10^{-11}	2.51665×10^{-10}	Yes
NUDT1	2.79468×10^{-10}	1.34145×10^{-9}	Yes
NSUN2	0.002675	0.003378	Yes
NCBP3	1.76262×10^{-5}	4.23030×10^{-5}	Yes
NCBP2	0.814540	0.814540	No
NCBP1	5.23996×10^{-9}	1.79656×10^{-8}	Yes
METTL1	3.05954×10^{-9}	1.22382×10^{-8}	Yes
LARP1	0.001511	0.002015	Yes
IFIT5	2.64779×10^{-4}	4.88823×10^{-4}	Yes
GEMIN5	3.31571×10^{-4}	5.68407×10^{-4}	Yes
EIF4E3	0.694601	0.724801	No
EIF4E	8.28925×10^{-11}	4.97355×10^{-10}	Yes
EIF4A1	1.20998×10^{-5}	3.22661×10^{-5}	Yes
EIF3D	0.018948	0.021654	Yes
DCP2	4.15186×10^{-4}	6.64297×10^{-4}	Yes
AGO2	5.89807×10^{-5}	1.28685×10^{-4}	Yes

Note: P values were adjusted using the Benjamini–Hochberg procedure across all 24 candidate genes. An adjusted *P* value < 0.05 was considered statistically significant. BH, Benjamini–Hochberg; PFAS, per- and polyfluoroalkyl substances. Values are rounded for display; significance was determined using unrounded adjusted P values.
