# Supplementary material for: Per- and Polyfluoroalkyl Substances and Papillary Thyroid Carcinoma: An Integrative Study of Bioinformatics, Epidemiological Associations, and In Vitro Responses
Source: Int J Mol Sci. 2026 Sep 17;27(18):8296. doi: 10.3390/ijms27188296 (PMC13607646; doi:10.3390/ijms27188296)
Supplement: Supplementary file 1 [file ijms-27-08296-s001.zip › Table S2.pdf]

Table S2. Distribution of serum PFAS concentrations among participants with normal weight

PFAS	LOD (ng/mL)	ALL (n =57)		Control (n = 38)		PTC (n = 19)		*P*
		Detection rate (%)	P_{50} (P_{25}, P_{75})	Detection rate (%)	P_{50} (P_{25}, P_{75})	Detection rate (%)	P_{50} (P_{25}, P_{75})	
9ClPF3ONS	0.02	91.2	0.21 (0.12, 0.38)	97.4	0.22 (0.15, 0.40)	78.9	0.14 (0.06, 0.24)	**0.018**
PFBS	0.02	29.8	0.02 (0.02, 0.02)	44.7	0.02 (0.02, 0.03)	0.0	0.02 (0.02, 0.02)	**0.004**
PFHxS	0.10	31.6	0.07 (0.04, 0.14)	36.8	0.08 (0.04, 0.15)	21.1	0.07 (0.04, 0.09)	0.263
PFOS	0.01	100.0	1.12 (0.66, 1.80)	100.0	1.24 (0.81, 1.96)	100.0	0.78 (0.46, 1.36)	0.039
PFUnA	0.01	100.0	0.26 (0.12, 0.39)	100.0	0.27 (0.13, 0.41)	100.0	0.21 (0.06, 0.36)	0.153
PFDA	0.10	73.7	0.19 (0.09, 0.30)	76.3	0.20 (0.12, 0.36)	68.4	0.16 (0.08, 0.24)	0.204
PFBA	0.10	42.1	0.09 (0.07, 0.13)	55.3	0.11 (0.09, 0.15)	15.8	0.07 (0.07, 0.08)	**<0.001**
PFDoA	0.01	59.6	0.01 (0.01, 0.03)	65.8	0.01 (0.01, 0.02)	47.4	0.01 (0.01, 0.03)	0.623
PFHpA	0.01	56.1	0.01 (0.01, 0.03)	60.5	0.01 (0.01, 0.03)	47.4	0.01 (0.01, 0.03)	0.799
PFNA	0.10	71.9	0.15 (0.07, 0.23)	76.3	0.16 (0.10, 0.24)	63.2	0.13 (0.05, 0.23)	0.388
PFOA	0.05	100.0	0.98 (0.63, 1.50)	100.0	0.98 (0.68, 1.37)	100.0	0.79 (0.47, 1.64)	0.703
PFPeA	0.01	100.0	0.28 (0.28, 0.28)	100.0	0.28 (0.28, 0.28)	100.0	0.28 (0.17, 0.28)	0.213
PFTeDA	0.01	100.0	0.17 (0.16, 0.17)	100.0	0.17 (0.16, 0.17)	100.0	0.17 (0.16, 0.17)	0.945
PFTrDA	0.01	68.4	0.03 (0.01, 0.04)	73.7	0.03 (0.01, 0.05)	57.9	0.01 (0.01, 0.04)	0.321

Note: PFAS concentrations are presented as detection rates (%) and median (P_{25}, P_{75}). Values below the limit of detection (LOD) were imputed as LOD/ √2. P values were calculated using the Mann‑Whitney U test. Bold values indicate statistical significance (P < 0.05). PTC, papillary thyroid carcinoma.
