# Supplementary material for: Per- and Polyfluoroalkyl Substances and Papillary Thyroid Carcinoma: An Integrative Study of Bioinformatics, Epidemiological Associations, and In Vitro Responses
Source: Int J Mol Sci. 2026 Sep 17;27(18):8296. doi: 10.3390/ijms27188296 (PMC13607646; doi:10.3390/ijms27188296)
Supplement: Supplementary file 1 [file ijms-27-08296-s001.zip › Table S3.pdf]

Table S3. Distribution of serum PFAS concentrations among overweight or obese participants

PFAS	LOD (ng/mL)	ALL (n =63)		Control (n = 22)		PTC (n = 41)		*P*
		Detection rate (%)	P_{50} (P_{25}, P_{75})	Detection rate (%)	P_{50} (P_{25}, P_{75})	Detection rate (%)	P_{50} (P_{25}, P_{75})	
9ClPF3ONS	0.02	93.7	0.19 (0.07, 0.33)	95.5	0.22 (0.13, 0.35)	92.7	0.18 (0.05, 0.30)	0.199
PFBS	0.02	14.3	0.02 (0.02, 0.02)	36.4	0.02 (0.02, 0.02)	2.4	0.02 (0.02, 0.02)	**0.021**
PFHxS	0.10	23.8	0.07 (0.06, 0.09)	22.7	0.07 (0.06, 0.09)	24.4	0.07 (0.05, 0.09)	0.594
PFOS	0.01	100.0	1.06 (0.53, 1.71)	100.0	1.21 (0.67, 1.64)	100.0	1.01 (0.52, 1.79)	0.705
PFUnA	0.01	98.4	0.20 (0.07, 0.30)	100.0	0.25 (0.15, 0.36)	97.6	0.12 (0.07, 0.26)	**0.027**
PFDA	0.10	65.1	0.15 (0.08, 0.22)	68.2	0.18 (0.09, 0.24)	63.4	0.13 (0.07, 0.22)	0.264
PFBA	0.10	28.6	0.07 (0.07, 0.10)	40.9	0.09 (0.07, 0.13)	22.0	0.07 (0.06, 0.10)	**0.005**
PFDoA	0.01	50.8	0.01 (0.01, 0.02)	68.2	0.01 (0.01, 0.02)	41.5	0.01 (0.01, 0.02)	0.061
PFHpA	0.01	60.3	0.01 (0.01, 0.02)	68.2	0.01 (0.01, 0.02)	56.1	0.01 (0.01, 0.02)	0.66
PFNA	0.10	60.3	0.12 (0.06, 0.18)	63.6	0.13 (0.07, 0.18)	58.5	0.12 (0.06, 0.17)	0.48
PFOA	0.05	100.0	0.89 (0.43, 1.22)	100.0	0.92 (0.63, 1.33)	100.0	0.85 (0.41, 1.15)	0.226
PFPeA	0.01	100.0	0.28 (0.17, 0.28)	100.0	0.28 (0.19, 0.28)	100.0	0.17 (0.17, 0.28)	**0.021**
PFTeDA	0.01	100.0	0.16 (0.16, 0.17)	100.0	0.16 (0.16, 0.17)	100.0	0.16 (0.16, 0.17)	0.099
PFTrDA	0.01	47.6	0.01 (0.01, 0.03)	72.7	0.02 (0.01, 0.04)	34.1	0.01(0.01, 0.02)	**0.007**

Note: PFAS concentrations are presented as detection rates (%) and median (P_{25}, P_{75}). Values below the limit of detection (LOD) were imputed as LOD/ √2. P values were calculated using the Mann – Whitney U test. Bold values indicate statistical significance ($P < 0.05$). PTC, papillary thyroid carcinoma.
