# Supplementary material for: Per- and Polyfluoroalkyl Substances and Papillary Thyroid Carcinoma: An Integrative Study of Bioinformatics, Epidemiological Associations, and In Vitro Responses
Source: Int J Mol Sci. 2026 Sep 17;27(18):8296. doi: 10.3390/ijms27188296 (PMC13607646; doi:10.3390/ijms27188296)
Supplement: Supplementary file 1 [file ijms-27-08296-s001.zip › Table S4.pdf]

Table S4. Associations between tertiles of ln-transformed PFAS concentrations and risk of papillary thyroid carcinoma

PFAS	Tertile 1	Tertile 2		Tertile 3		P for trend	Trend_FDR
		OR(95%CI)	*P*	OR(95%CI)	*P*		
9Cl-PF3ONS							
Model 1	Reference	0.44(0.18,1.07)	0.074	0.36(0.14,0.88)	**0.027**	**0.022**	0.051
Model 2	Reference	0.34(0.11,1.02)	0.057	0.27(0.09,0.77)	**0.017**	**0.013**	**0.036**
PFHxS							
Model 1	Reference	1.22(0.51,2.98)	0.653	0.60(0.25,1.46)	0.264	0.248	0.316
Model 2	Reference	1.17(0.40,3.37)	0.777	0.50(0.16,1.51)	0.226	0.234	0.303
PFOS							
Model 1	Reference	0.74(0.30,1.78)	0.502	0.55(0.22,1.32)	0.181	0.182	0.316
Model 2	Reference	0.70(0.24,2.04)	0.510	0.31(0.10,0.92)	**0.041**	**0.044**	0.089
PFUnA							
Model 1	Reference	0.29(0.11,0.71)	**0.008**	0.29(0.11,0.71)	**0.008**	**0.005**	**0.013**
Model 2	Reference	0.18(0.05,0.55)	**0.004**	0.22(0.07,0.70)	**0.012**	**0.005**	**0.025**
PFDA							
Model 1	Reference	0.90(0.37,2.19)	0.822	0.44(0.18,1.08)	0.075	0.092	0.184
Model 2	Reference	1.19(0.38,3.77)	0.765	0.32(0.10,0.95)	**0.046**	0.076	0.132
PFBA							
Model 1	Reference	0.24(0.09,0.61)	**0.004**	0.11(0.04,0.29)	**<0.001**	**<0.001**	**<0.001**
Model 2	Reference	0.22(0.06,0.72)	**0.015**	0.10(0.03,0.32)	**<0.001**	**<0.001**	**0.003**
PFDoA							
Model 1	Reference	1.11(0.46,2.67)	0.823	0.91(0.37,2.18)	0.823	0.827	0.827
Model 2	Reference	1.31(0.43,3.98)	0.637	0.87(0.30,2.53)	0.798	0.819	0.819
PFHpA							
Model 1	Reference	1.00(0.41,2.41)	1.000	0.74(0.31,1.78)	0.503	0.521	0.561
Model 2	Reference	0.74(0.25,2.12)	0.573	0.76(0.25,2.26)	0.625	0.598	0.644
PFNA							
Model 1	Reference	0.67(0.27,1.61)	0.371	0.61(0.25,1.46)	0.265	0.247	0.316

Model 2	Reference	0.65(0.22,1.93)	0.441	0.50(0.17,1.48)	0.219	0.209	0.303
PFOA							
Model 1	Reference	0.44(0.18,1.08)	0.076	0.67(0.27,1.61)	0.370	0.309	0.361
Model 2	Reference	0.32(0.10,0.93)	**0.041**	0.67(0.23,1.95)	0.467	0.315	0.367
PFTeDA							
Model 1	Reference	3.89(1.56,10.16)	**0.004**	3.16(1.28,8.15)	**0.015**	0.247	0.316
Model 2	Reference	3.84(1.26,12.57)	**0.021**	3.28(1.09,10.45)	**0.038**	0.238	0.303
PFTrDA							
Model 1	Reference	0.81(0.33,1.99)	0.648	0.26(0.10,0.64)	**0.004**	**0.005**	**0.013**
Model 2	Reference	0.61(0.20,1.82)	0.382	0.21(0.06,0.66)	**0.010**	**0.010**	**0.036**

Note: OR, odds ratio; CI, confidence interval. Model 1 was unadjusted. Model 2 was adjusted for age, sex, BMI, residence, smoking status, and alcohol cons umption. PFAS concentrations were categorized into tertiles based on their distr ibutions among controls. Bold values indicate statistical significance (*P* < 0.05).
