# Supplementary material for: Per- and Polyfluoroalkyl Substances and Papillary Thyroid Carcinoma: An Integrative Study of Bioinformatics, Epidemiological Associations, and In Vitro Responses
Source: Int J Mol Sci. 2026 Sep 17;27(18):8296. doi: 10.3390/ijms27188296 (PMC13607646; doi:10.3390/ijms27188296)
Supplement: Supplementary file 1 [file ijms-27-08296-s001.zip › Table S5.pdf]

Table S5. Associations between tertiles of ln-transformed PFAS concentrations and risk of papillary thyroid carcinoma among women

PFAS	Tertile 1	Tertile 2		Tertile 3		*P* for trend
		OR(95%CI)	*P*	OR(95%CI)	*P*	
9ClPF3ONS	Reference	0.75(0.18,3.22)	0.701	0.15(0.03,0.74)	**0.020**	**0.050**
PFHxS	Reference	1.26(0.28,5.55)	0.764	0.20(0.04,0.93)	**0.041**	0.057
PFOS	Reference	1.12(0.27,4.55)	0.878	0.42(0.10,1.81)	0.242	0.389
PFUnA	Reference	0.22(0.05,0.93)	**0.039**	0.36(0.08,1.71)	0.197	0.114
PFDA	Reference	1.39(0.34,5.68)	0.644	0.69(0.16,2.95)	0.615	0.641
PFBA	Reference	0.04(0.01,0.30)	**0.002**	0.03(0.00,0.23)	**0.001**	**0.002**
PFDoA	Reference	0.41(0.10,1.73)	0.225	0.78(0.18,3.32)	0.733	0.463
PFHpA	Reference	2.19(0.51,9.46)	0.294	1.31(0.30,5.76)	0.722	0.561
PFNA	Reference	0.83(0.21,3.26)	0.791	1.09(0.25,4.71)	0.912	0.932
PFOA	Reference	0.64(0.16,2.55)	0.530	0.71(0.17,3.04)	0.643	0.806
PFTeDA	Reference	0.59(0.14,2.54)	0.479	0.66(0.16,2.79)	0.570	0.758
PFTrDA	Reference	1.14(0.27,4.90)	0.859	0.53(0.09,2.99)	0.468	0.573

Note: OR, odds ratio; CI, confidence interval. Models were adjusted for age, residence, smoking status, and alcohol consumption, with sex or BMI omitted as appropriate in stratified analyses. Bold values indicate statistical significance ($P < 0.05$).
