# Supplementary material for: Per- and Polyfluoroalkyl Substances and Papillary Thyroid Carcinoma: An Integrative Study of Bioinformatics, Epidemiological Associations, and In Vitro Responses
Source: Int J Mol Sci. 2026 Sep 17;27(18):8296. doi: 10.3390/ijms27188296 (PMC13607646; doi:10.3390/ijms27188296)
Supplement: Supplementary file 1 [file ijms-27-08296-s001.zip › Table S6.pdf]

Table S6. Associations between tertiles of ln-transformed PFAS concentrations and risk of papillary thyroid carcinoma among participants with normal weight

PFAS	Tertile 1	Tertile 2		Tertile 3		*P* for trend
		OR(95%CI)	*P*	OR(95%CI)	*P*	
9ClPF3ONS	Reference	0.35(0.07,1.74)	0.199	0.06(0.01,0.42)	**0.005**	**0.020**
PFHxS	Reference	2.39(0.42,13.53)	0.325	0.18(0.03,1.06)	0.058	**0.045**
PFOS	Reference	0.55(0.12,2.45)	0.431	0.12(0.02,0.86)	**0.034**	0.103
PFUnA	Reference	0.08(0.01,0.54)	**0.010**	0.12(0.02,0.69)	**0.017**	**0.018**
PFDA	Reference	0.99(0.20,4.96)	0.991	0.25(0.05,1.26)	0.094	0.184
PFBA	Reference	0.04(0.00,0.42)	**0.007**	0.02(0.00,0.17)	**0.001**	**0.002**
PFDoA	Reference	0.56(0.11,2.80)	0.482	0.61(0.13,2.87)	0.528	0.733
PFHpA	Reference	0.65(0.13,3.29)	0.598	0.58(0.12,2.89)	0.505	0.787
PFNA	Reference	0.51(0.10,2.48)	0.402	0.43(0.09,1.97)	0.275	0.508
PFOA	Reference	0.25(0.05,1.28)	0.096	0.62(0.13,2.97)	0.550	0.242
PFTeDA	Reference	0.33(0.06,1.74)	0.192	0.30(0.06,1.59)	0.157	0.305
PFTrDA	Reference	1.29(0.23,7.44)	0.774	0.42(0.06,3.02)	0.391	0.356

Note: OR, odds ratio; CI, confidence interval. Models were adjusted for age, residence, smoking status, and alcohol consumption. Bold values indicate statistical significance ($P < 0.05$). PTC, papillary thyroid carcinoma.
