# Supplementary material for: Per- and Polyfluoroalkyl Substances and Papillary Thyroid Carcinoma: An Integrative Study of Bioinformatics, Epidemiological Associations, and In Vitro Responses
Source: Int J Mol Sci. 2026 Sep 17;27(18):8296. doi: 10.3390/ijms27188296 (PMC13607646; doi:10.3390/ijms27188296)
Supplement: Supplementary file 1 [file ijms-27-08296-s001.zip › Table S7.pdf]

Table S7. Associations between tertiles of ln-transformed PFAS concentrations and risk of papillary thyroid carcinoma among overweight or obese participants

PFAS	Tertile 1	Tertile 2		Tertile 3		*P* for tren d
		OR(95%CI)	*P*	OR(95%CI)	*P*	
9ClPF3ONS	Reference	0.19(0.03,1.13)	0.068	0.35(0.07,1.79)	0.206	0.169
PFHxS	Reference	0.95(0.18,5.04)	0.953	1.50(0.21,10.9 6)	0.689	0.872
PFOS	Reference	0.49(0.09,2.75)	0.419	0.53(0.11,2.42)	0.409	0.628
PFUnA	Reference	0.22(0.04,1.24)	0.086	0.14(0.02,0.97)	**0.047**	0.103
PFDA	Reference	1.85(0.36,9.36)	0.459	0.22(0.03,1.49)	0.120	0.148
PFBA	Reference	0.39(0.07,2.10)	0.273	0.16(0.03,1.05)	0.056	0.155
PFDoA	Reference	0.21(0.03,1.46)	0.113	0.21(0.03,1.63)	0.135	0.241
PFHpA	Reference	1.06(0.23,4.90)	0.937	1.33(0.22,8.15)	0.759	0.951
PFNA	Reference	1.08(0.24,4.95)	0.921	0.34(0.06,2.01)	0.232	0.410
PFOA	Reference	0.19(0.04,1.04)	0.055	0.39(0.07,2.19)	0.286	0.149
PFTeDA	Reference	0.11(0.02,0.65)	**0.015**	0.60(0.10,3.62)	0.574	**0.047**
PFTrDA	Reference	1.13(0.17,7.62)	0.899	0.13(0.02,1.05)	0.056	0.055

Note: OR, odds ratio; CI, confidence interval. Models were adjusted for age, residence, smoking status, and alcohol consumption. Bold values indicate statistical significance ($P < 0.05$). PTC, papillary thyroid carcinoma.
