# Supplementary material for: Per- and Polyfluoroalkyl Substances and Papillary Thyroid Carcinoma: An Integrative Study of Bioinformatics, Epidemiological Associations, and In Vitro Responses
Source: Int J Mol Sci. 2026 Sep 17;27(18):8296. doi: 10.3390/ijms27188296 (PMC13607646; doi:10.3390/ijms27188296)
Supplement: Supplementary file 1 [file ijms-27-08296-s001.zip › Table S8.pdf]

Table S8 Mass spectrometry analysis parameters for target compounds

PFAS	Full Name	Precursor Ion	Product Ion	Ionization Mode
11Cl-PF3OUdS	Potassium 11-chloroeicosafluoro-3-oxaundecane-1-sulfonate	531.00	351.00	Negative
9Cl-PF3ONS	Potassium 9-chlorohexadecafluoro-3-oxanonane-1-sulfonate	631.00	451.00	Negative
PFBS	Potassium perfluoro-1-butanesulfonate	298.90	80.00	Negative
PFHpS	Sodium perfluoro-1-heptanesulfonate	448.90	79.80	Negative
PFHxS	Sodium perfluoro-1-hexanesulfonate	398.90	80.00	Negative
PFOS	Sodium perfluoro-1-octanesulfonate	498.90	80.00	Negative
PFPeS	Sodium perfluoro-1-pentanesulfonate	348.90	80.00	Negative
PFUnA	perfluoroundecanoic acid	562.90	518.80	Negative
PFDA	Perfluorodecanoic acid	512.90	469.10	Negative
PFBA	Heptafluorobutyric acid	212.80	168.90	Negative
PFDoA	Perfluoro-n-dodecanoic acid	613.00	569.00	Negative
PFHpA	Perfluoroheptanoic acid	363.00	319.10	Negative
PFNA	Perfluorononanoic acid	462.80	419.00	Negative
PFOA	Perfluorooctanoic acid	413.00	368.90	Negative
PFPeA	Perfluoro-n-pentanoic acid	263.00	218.90	Negative
PFTeDA	Perfluoro-n-tetradecanoic acid	713.00	669.00	Negative
PFTrDA	Perfluoro-n-tridecanoic acid	663.00	619.00	Negative

Note: UHPLC–MS/MS analysis was performed in multiple reaction monitoring (MRM) mode. All compounds were detected using negative electrospray ionization (ESI−).
