# Supplementary material for: Per- and Polyfluoroalkyl Substances and Papillary Thyroid Carcinoma: An Integrative Study of Bioinformatics, Epidemiological Associations, and In Vitro Responses
Source: Int J Mol Sci. 2026 Sep 17;27(18):8296. doi: 10.3390/ijms27188296 (PMC13607646; doi:10.3390/ijms27188296)
Supplement: Supplementary file 1 [file ijms-27-08296-s001.zip › Table S9.pdf]

Table S9. Molecular characteristics and analytical performance of the target PFAS compounds

PFAS compounds	Molecular formula	LOD（ng/mL）	LOQ（ng/mL）	ULOQ（ng/mL）	R^2	Calibration (ng/mL)	Recovery (%)	Precision (RSD, %)
11Cl-PF3OUdS	$C_{10}HClF_{20}O_4S$	0.02	0.05	200.00	0.9986	20.0	106.44	3.22
9Cl-PF3ONS	$C_9HClF_{16}O_4S$	0.02	0.05	100.00	0.9989	20.0	98.70	5.62
PFBS	$C_4HF_9O_3S$	0.02	0.05	100.00	0.9992	20.0	96.59	5.71
PFHpS	$C_7HF_{15}O_3S$	0.02	0.05	200.00	0.9991	20.0	94.73	7.88
PFHxS	$C_6HF_{13}O_3S$	0.10	0.20	200.00	0.9993	20.0	91.36	14.13
PFOS	$C_8HF_{17}O_3S$	0.01	0.02	100.00	0.9999	20.0	99.64	3.08
PFPeS	$C_5HF_{11}O_3S$	0.01	0.02	100.00	0.9994	20.0	96.78	3.78
PFUnA	$C_{11}HF_{21}O_2$	0.01	0.02	100.00	0.9998	20.0	112.22	6.53
PFDA	$C_{10}HF_{19}O_2$	0.10	0.20	200.00	0.9982	20.0	105.12	4.81
PFBA	$C_4HF_7O_2$	0.10	0.20	25.00	0.9963	20.0	99.99	2.97
PFDoA	$C_{12}HF_{23}O_2$	0.01	0.02	25.00	0.9999	20.0	117.53	3.09
PFHpA	$C_7HF_{13}O_2$	0.01	0.02	200.00	0.9991	20.0	98.67	3.91
PFNA	$C_9HF_{17}O_2$	0.10	0.20	100.00	0.9979	20.0	100.10	6.76
PFOA	$C_8HF_{15}O_2$	0.05	0.10	50.00	0.9983	20.0	102.32	2.82
PFPeA	$C_5HF_9O_2$	0.39	0.78	200.00	0.9994	20.0	98.39	3.07
PFTeDA	$C_{14}HF_{27}O_2$	0.39	0.78	200.00	0.9989	20.0	96.82	4.72
PFTrDA	$C_{13}HF_{25}O_2$	0.01	0.02	50.00	0.9999	20.0	116.20	3.90

Note: LOD, limit of detection; LOQ, limit of quantification; ULOQ, upper limit of quantification; R^2, coefficient of determination.
